# Adverse events profiles of pemetrexed: a Food and Drug Administration Adverse Event Reporting System

**DOI:** 10.3389/fmed.2026.1750861

**Published:** 2026-04-15

**Authors:** Lei Wang, JianHang Su, Chuliang Huang, Yukun Yang, Yuanqing Xu

**Affiliations:** 1 The Sixth People's Hospital of Huizhou, Huizhou, China; 2 Sun Yat-Sen Memorial Hospital, Guangzhou, China

**Keywords:** disproportionality analysis, FDA Adverse Event Reporting System (FAERS), FDA, pemetrexed, pharmacovigilance

## Abstract

**Background:**

Pemetrexed is a folate-pathway-targeting antineoplastic agent widely used in non-squamous non-small cell lung cancer (NSCLC) and malignant pleural mesothelioma. We used the US Food and Drug Administration Adverse Event Reporting System (FAERS) to characterize pemetrexed-associated adverse events to inform clinical safety monitoring.

**Methods:**

Pemetrexed-related reports were retrieved from the FAERS database from first quarter of 2004 through the fourth quarter of 2024. All reports were coded using the Medical Dictionary for Regulatory Activities (MedDRA) terminology. We performed descriptive analyses and disproportionality analyses using reporting odds ratio (ROR), proportional reporting ratio (PRR), empirical Bayes geometric mean (EBGM), and Bayesian confidence propagation neural network (BCPNN; information component [IC]) to identify safety signals. Statistical significance was defined as ROR >1 with the lower 95% confidence interval (CI) >1.

**Results:**

We identified 27,098 pemetrexed-associated reports in the FAERS database (2004–2024), in which pemetrexed was designated as the primary suspect drug. Signal detection based on these reports identified 653 significant signals of disproportionate reporting (SDR) spanning 27 System Organ Classes (SOCs). The strongest signals occurred in Blood and Lymphatic Disorders (ROR = 7.31, 95% CI = 7.16–7.47) and Endocrine disorders (ROR = 4.09, 95% CI = 3.83–4.37), the latter representing unlabeled findings. In addition, we detected previously unlisted AEs, including colitis, oral lesions, immune-mediated pancreatitis, pulmonary toxicity, lung consolidation and tubulointerstitial nephritis. Sex stratification revealed male predominance in respiratory toxicity vs. female predisposition to renal injury. A bimodal time-to-onset distribution was observed: 52.57% of AEs occurred within 0–30 days post-treatment, with a secondary peak (6.63%) at 181–360 days.

**Conclusion:**

This large pharmacovigilance study identifies signals of disproportionate reporting (SDRs) consistent with pemetrexed's established toxicities (e.g., myelosuppression, nephrotoxicity) and detects additional unlabeled safety signals, particularly immune-mediated endocrine, renal, and dermatologic manifestations. The bimodal onset pattern of these SDRs highlights clinically relevant monitoring windows, and observed sex- and age-related differences in SDR reporting suggest a need for individualized risk mitigation. These findings underscore the importance of vigilant surveillance for potential immune-mediated toxicities and adherence to vitamin supplementation protocols to manage hematologic risk. Prospective studies are needed to validate these reporting associations and clarify potential causality for these novel signals.

## 1 Introduction

Pemetrexed, a multi-targeted antifolate agent, received initial U.S. Food and Drug Administration (FDA) regulatory approval on February 4, 2004, in combination with cisplatin for the treatment of unresectable malignant pleural mesothelioma (MPM) or for patients who were not candidates for curative surgery (1). Subsequently in August 2004, the FDA expanded its therapeutic indications to include second-line monotherapy for locally advanced or metastatic non-small cell lung carcinoma (NSCLC) [ICD-10: C34.90] (2). Over time, pemetrexed has demonstrated clinically significant efficacy in the management of diverse malignant neoplasms, including breast cancer (3), pancreatic cancer (4), ovarian cancer (5), cervical cancer (6), and colorectal cancer (7). Pemetrexed generally has a favorable toxicity profile and is generally well-tolerated. However, its dose-limiting and treatment-limiting toxicities—such as mucositis, dermatological reaction, fatigue, nephrotoxicity, and neutropenia—remain clinically important. Notably, renal toxicities and hematological toxicities continue to be common causes for treatment discontinuation (8). Prompt identification and proactive management of potential adverse events (AEs) enhance both treatment adherence and quality of life (QoL). Currently, no comprehensive studies have analyzed pemetrexed-associated adverse events using large-scale real-world data.

Pharmacovigilance, first established through the US Drug Efficacy Amendments (1962; Kefauver-Harris Amendments) (9), was formally defined by the WHO in 2002 as “the science and activities relating to the detection, assessment, understanding, and prevention of adverse drug reactions (ADRs) or any other drug-related problems.” The US Food and Drug Administration Adverse Event Reporting System (FAERS), a spontaneous reporting system database, provides FDA-curated real-world data on post-marketing safety reports and individual case safety reports (ICSRs) for pharmaceuticals and therapeutic biological products. Leveraging the FAERS database, this study evaluated and analyzed safety signals of pemetrexed-associated adverse events (AEs) to inform evidence-based clinical decision-making.

## 2 Methods

### 2.1 Data sources

This study analyzed AEs associated with pemetrexed as reported in FAERS database. Data from the first quarter of 2004 to the fourth quarter of 2024, the FAERS online portal (https://www.fda.gov/drugs/drug-approvals-and-databases/fda-adverse-event-reporting-system-faers-database). FAERS provides demographic and administrative information (DEMO), drug information (DRUG), adverse event details (REAC), patient outcomes (OUTC), report sources (RPSR), treatment start and end dates (THER), and indications for drug use (INDI). Inclusion criteria were defined as reports where pemetrexed was designated as the primary suspect (PS) or secondary suspect (SS) drug; exclusion criteria included extreme value records, non-adverse events reports, duplicate entries, and cases listed in FDA's deleted cases file.

### 2.2 AEs and drug identification

All adverse events (AEs) in the FAERS database were coded using the Medical Dictionary for Regulatory Activities (MedDRA) preferred terms (PTs). MedDRA classifies AEs into five hierarchical levels: PTs, high-level terms (HLTs), high-level group terms (HLGTs), System Organ Classes (SOCs), and lowest level terms (LLTs). We focused on PTs and SOCs because PTs provide the most clinically interpretable and specific adverse-event labels for signal detection, whereas SOCs summarize organ-system patterns for high-level safety profiling. Higher MedDRA levels (e.g., HLT/HLGT) were not prioritized because they aggregate overlapping PTs and may reduce clinical interpretability without adding meaningful value in spontaneous-reporting data. No PTs were manually reviewed or excluded based on clinical relevance; all PTs were included and screened according to predefined statistical criteria.

To analyzed pemetrexed-associated AEs We identified relevant reports using both the brand name (ALIMTA) and generic name (PEMETREXED). Drugs were classified as primary suspect (PS), secondary suspect (SS), concomitant, or interacting agents. To ensure robustness and reduce confounding bias, only records where pemetrexed was designated as the unique PS drug from the DRUG files were included; reports with multiple drugs marked as PS were excluded at the initial extraction stage. For reports with pemetrexed as the unique PS (accompanied by SS or concomitant drugs), such records were retained.

Duplicate case reports were removed according to FDA recommendations. For cases with identical CASE IDs, we retained the report with the most recent FDA submission date (FDA_DT). When CASE IDs and FDA_DT matched, the record with the higher PRIMARY ID was selected. In addition to duplicate reports and FDA deleted cases, the following records were also excluded from the initial PS-designated reports to obtain the final 27,098 valid reports: (1) Extreme value records with logically inconsistent timelines or unverifiable AE severity; (2) Non-adverse event reports such as administrative entries.

Additionally, cases listed in the deleted cases files were excluded from analysis. Inclusion/exclusion criteria were pre-specified before data analysis, to avoid selective reporting bias.

### 2.3 Subgroup analysis

Subgroup analyses evaluated association between pemetrexed and adverse events (AEs), stratified by sex, age categories (< 18, 18–65, or ≥65 years), and reporter type (healthcare professionals vs. non-healthcare professionals).

### 2.4 Sensitivity analysis

To assess robustness under reduced co-medication complexity, we conducted a sensitivity analysis by excluding reports that also listed cisplatin, carboplatin, pembrolizumab, or bevacizumab. We also defined positive signals based on pre-specified statistical thresholds (Supplementary Table S1) and reported the resulting signals at the PT and SOC levels. No additional “clinical prioritization” step beyond these predefined criteria was performed. This analysis aimed to confirm key signal robustness of in a simplified co-medication setting, not to establish causal attribution.

### 2.5 Statistical analysis

Disproportionality analysis is a core pharmacovigilance method for identifying drug-related adverse events (AEs), used to assess the statistical association between pemetrexed and all reported AEs. To assess signal strength, we applied four disproportionality approaches: reporting odds ratio (ROR), proportional reporting ratio (PRR), empirical Bayes geometric mean (EBGM), and Bayesian confidence propagation neural network (BCPNN; information component [IC]). A positive safety signal was defined as ≥2 of the four methods (ROR, PRR, EBGM, and IC) indicating a significant signal.

**Figure 1:**
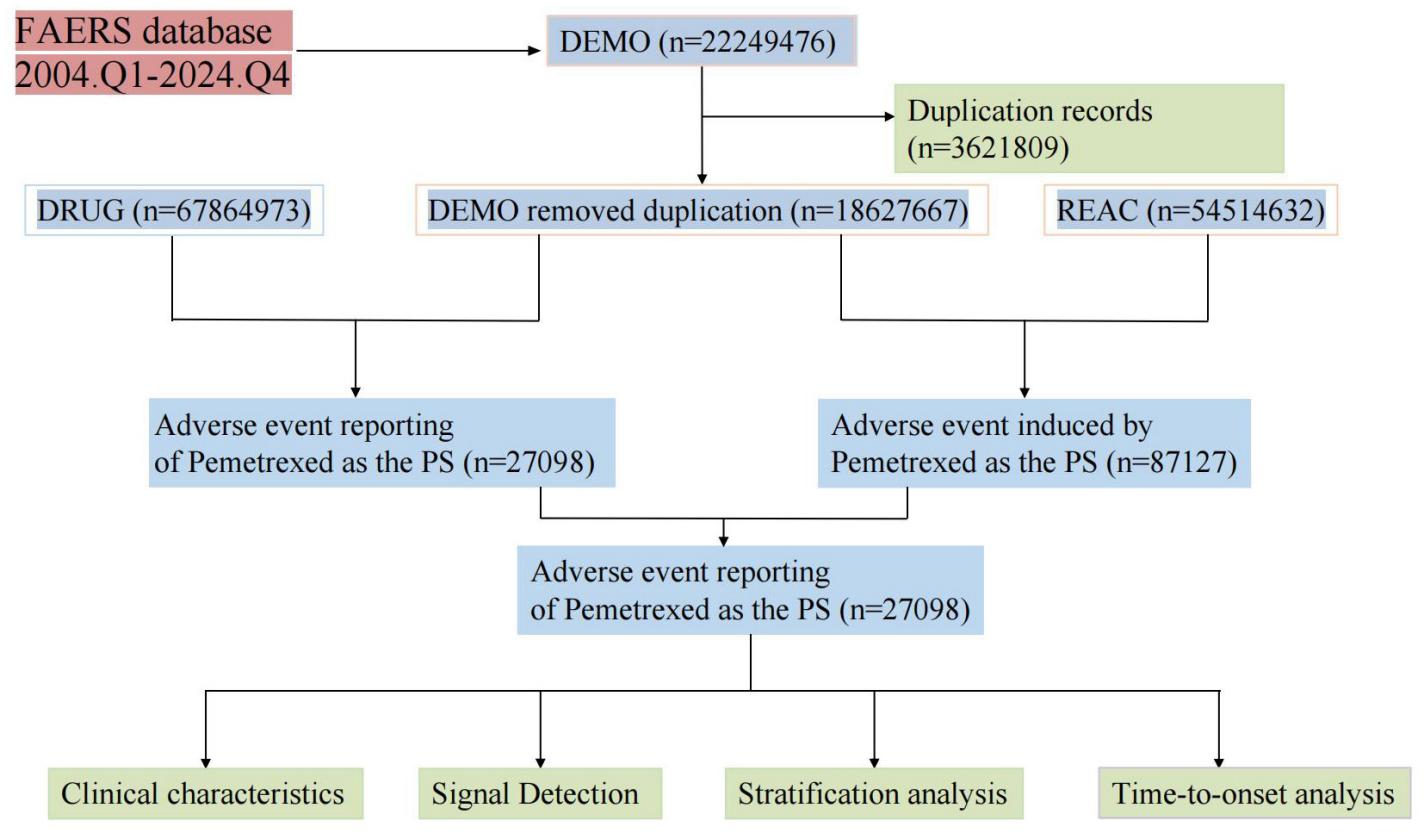
The flowchart illustrating the selection process of pemetrexed-associated AEs from the FAERS database.

The multi-step data processing workflow is summarized in Figure 1. We calculated ROR and PRR as frequentist measures and EBGM and IC as Bayesian measures to evaluate significant drug-AE associations. Detailed equations and criteria for these algorithms are provided in Supplementary Table S1. AEs meeting the pre-specified signal thresholds in ≥2 of the 4 methods (including at least one frequentist and one Bayesian metric) were classified as positive signals. For signal prioritization in the main text, we further required ≥100 reports per PT to enhance robustness; signals with fewer reports were considered exploratory and are provided in Supplementary material. ROR features straightforward computation and remains unaffected by non-selective underreporting. BCPNN, implementing Bayesian principles on 2 × 2 contingency tables, demonstrates superior performance in early AE signal detection. The key methodological difference is that ROR uses a frequentist framework, while IC is Bayesian. To address inconsistencies in the FAERS database, we manually matched drug trade names (DRUGNAME) with active ingredients (PROD_AI). AE occurrence dates (EVENT_DT) and pemetrexed initiation dates (START_DT) were extracted from the REAC files. Time-to-onset was calculated as: EVENT_DT – START_DT. All data processing and statistical analyses were performed using R software (R Foundation for Statistical Computing, Vienna, Austria; version 4.0.2; released on 22 June 2022).

## 3 Results

### 3.1 Descriptive analysis

**Figure 2:**
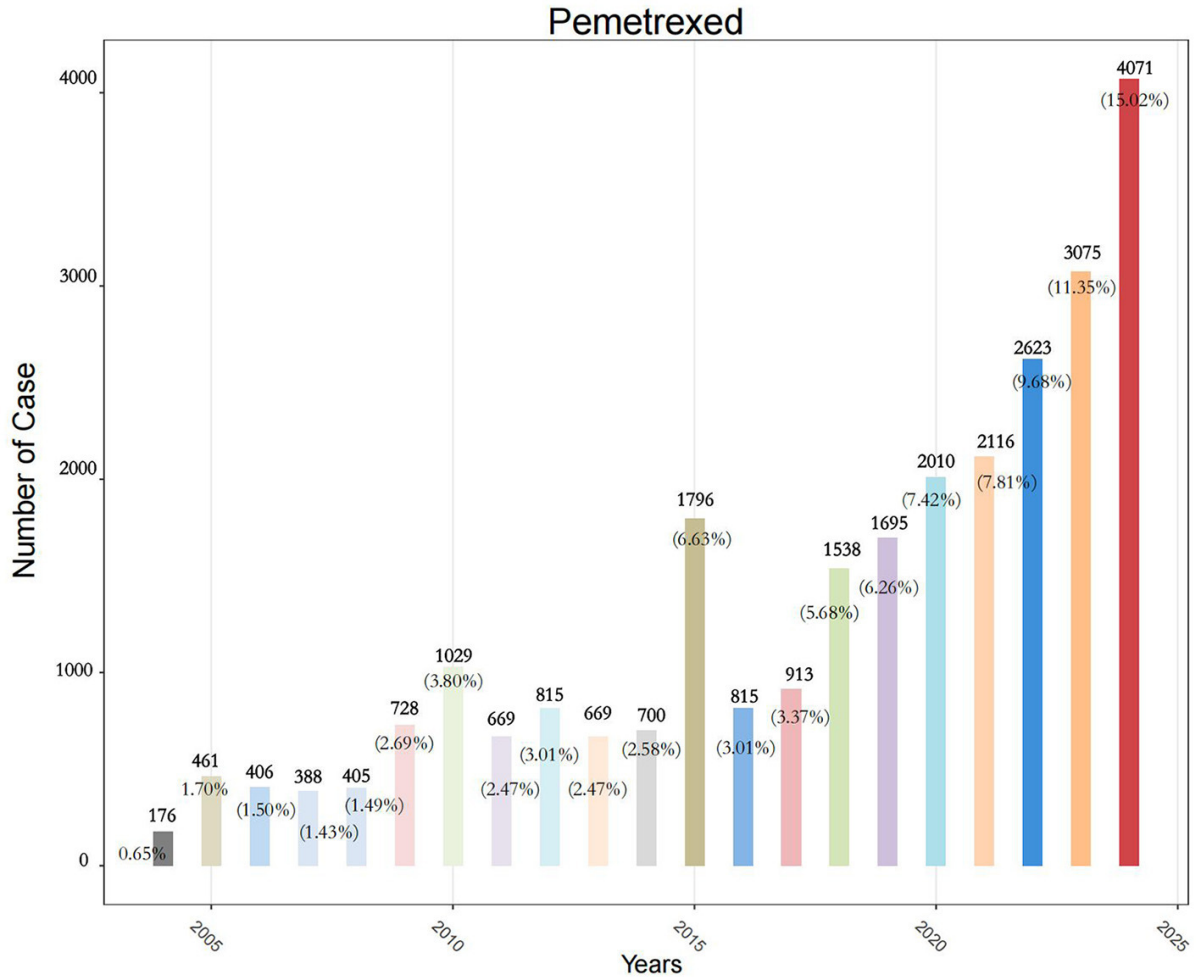
Temporal distribution of pemetrexed-associated adverse event reports in FAERS database (2004–2024).

A review of the FAERS yielded a total of 22,249,476 reports from the first quarter of 2004 to the fourth quarter of 2024, after eliminating duplicates. We identified 27,098 reports where pemetrexed was the unique PS drug. After applying the pre-specified exclusion criteria (extreme values, non-adverse event reports, duplicates, FDA deleted cases), 27,098 valid reports were retained for all subsequent statistical analyses. From 2004 to 2017, approximately 400 to 1,000 AE reports were documented annually. A notable increase to 1,000–4,000 reports per year was observed starting from 2018 (Figure 2). The clinical characteristics of these pemetrexed-associated AE reports are detailed in Table 1. Among the reports, males predominated (14,860 cases; 54.8%) over females (9,655 cases; 35.6%). Reporting frequencies were comparable between the 18–64 age group (37.1%) and the 65–85 cohort (38.1%), with 24.1% lacking age specification. The top five reporting countries/regions were the United States (23.4%), France (18.8%), Japan (10.7%), Germany (7.3%), and Italy (6.5%). Consumers submitted 22.4% of reports, whereas healthcare professionals (including physicians, other healthcare professionals, pharmacists, and registered nurses) accounted for 37.2%. The primary therapeutic indication was malignant lung neoplasms (65.3%), followed by mesothelioma. AE outcomes included hospitalization (34%), death, life-threatening events, and disabling conditions (hospitalization included prolonged stays); hospitalization was the most frequent serious outcome.

**Table 1:** Clinical characteristics of pemetrexed-associated reports from the FAERS database (2004Q1–2024Q4).

Characteristics	Numbers (*N*)	Case proportion (%)
Number of events	27,098	
Gender		
Male	14,860	54.80
Female	9,655	35.60
Unknown	2,583	9.50
Age		
< 18	73	0.30
18–64	10,043	37.10
65–85	10,336	38.10
>85	106	0.40
Miss	6,540	24.10
Reported countries (top 10)		
United States	6,349	23.40
France	5,094	18.80
Japan	2,886	10.70
Germany	1,977	7.30
Italy	1,756	6.50
China	1,482	5.50
United Kingdom	960	3.50
Spain	776	2.90
Canada	627	2.30
Netherlands	526	1.90
Reported person		
Physician	1,761	6.50
Consumer	6,074	22.40
Health professional	3,653	13.50
Other Health-professional	2,879	10.60
Pharmacist	1,761	6.50
Registered nurse	19	0.10
Miss	540	2.00
Indication (top 2)		
Lung neoplasm malignant	17,640	65.3
Mesothelioma	1,725	6.00
Serious outcomes		
Hospitalization	9,224	34.0
Death	5,010	18.5
Life threatening	1,393	5.10
Disability	240	0.90
Other serious medical events	7,945	29.3

### 3.2 Signals detection based on SOC levels

**Figure 3:**
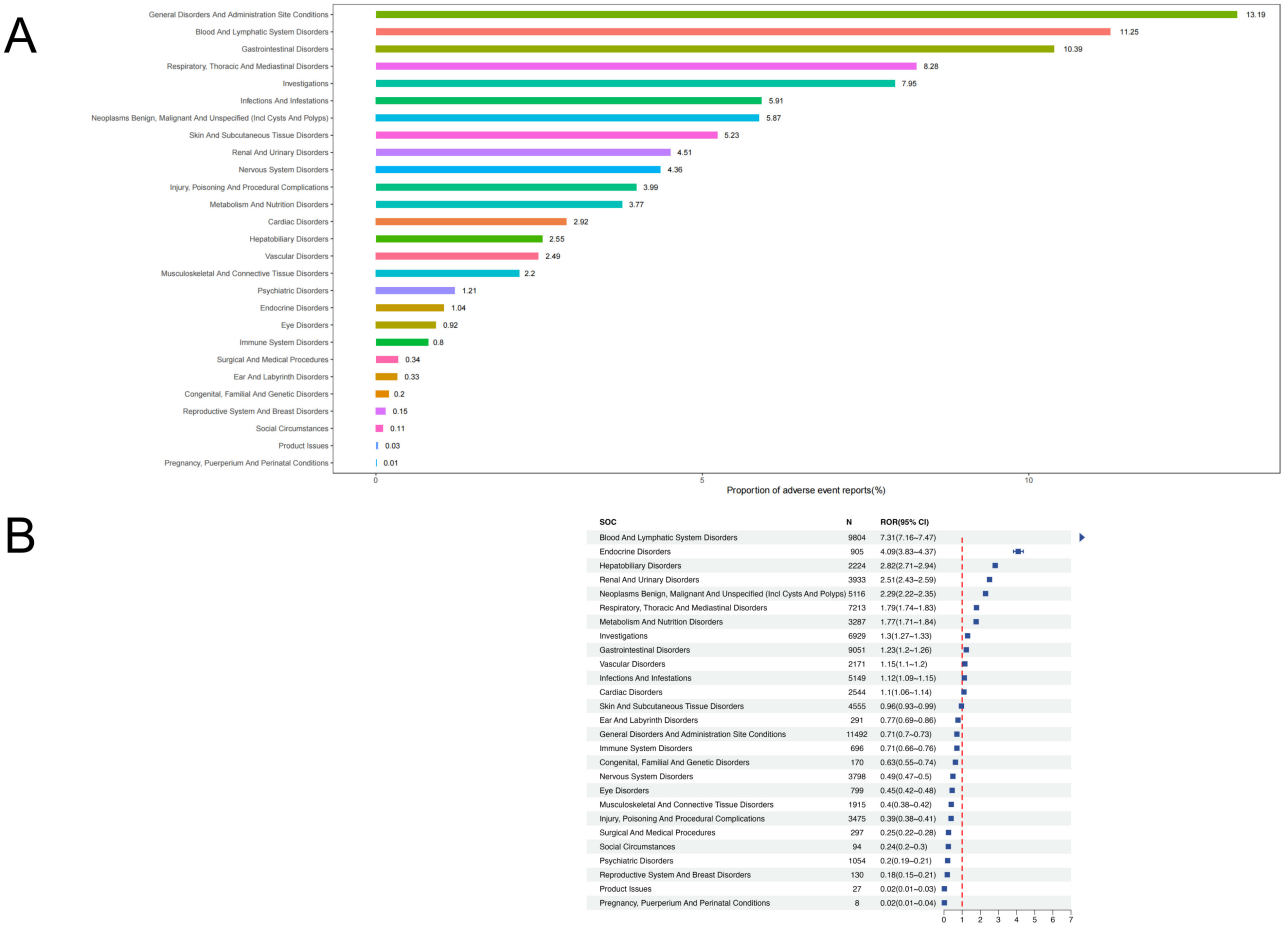
**(A)** Proportional distribution of pemetrexed-associated adverse events; **(B)** Signal strength of pemetrexed AEs across System Organ Classes (SOC) in the FAERS database.

Pemetrexed-associated AEs spanned 27 organ systems (Table 2). The highest proportions of adverse events were observed for the following five categories: General disorders and administration site conditions (13.13%), Blood and lymphatic system disorders (11.25%), Gastrointestinal disorders (10.39%), Respiratory, thoracic and mediastinal disorders (8.28%), and investigations (7.95%) (Figure 3A). Disproportionality analyses were conducted using four methods (ROR, PRR, EBGM, and IC/BCPNN) to quantify signal strength. For clarity of presentation, SOC-level signal strength is summarized using ROR (with 95% CI) in the main figures, while concordance across methods is provided in Supplementary Table S2. A statistically significant positive signal was defined as ROR >1 with the lower 95% confidence interval (95% CI) >1. The signal strengths and number of reports for pemetrexed-related AEs are presented in Figure 3B. Specifically, blood and lymphatic system disorders showed the strongest signal (*n* = 9,804, ROR = 7.31, 95% CI = 7.16–7.47), followed by endocrine disorders (*n* = 905, ROR = 4.09, 95% CI = 3.83–4.37), hepatobiliary disorders (*n* = 2,224, ROR = 2.82, 95% CI = 2.71–2.94), renal and urinary disorders (*n* = 3,933, ROR = 2.51, 95% CI = 2.43–2.59), neoplasms benign, malignant and unspecified (incl cysts And polyps) (*n* = 5,116, ROR = 2.29, 95% CI = 2.22–2.35), respiratory, thoracic and mediastinal disorders (*n* = 7,213, ROR = 1.79, 95% CI = 1.74–1.83), metabolism and nutrition disorders (*n* = 3,287, ROR = 1.77, 95% CI = 1.71–1.84), investigations (*n* = 6,929, ROR = 1.3, 95% CI = 1.27–1.33), gastrointestinal disorders (*n* = 9,051, ROR = 1.23, 95% CI = 1.2–1.26), vascular disorders (*n* = 2,171, ROR = 1.15, 95% CI = 1.1–1.2), infections and infestations (*n* = 5,149, ROR = 1.12, 95% CI = 1.09–1.15) and cardiac disorders (*n* = 2,544, ROR = 1.1, 95% CI = 1.06–1.14).

**Table 2:** Signal strength of pemetrexed AEs across System Organ Classes (SOC) in the FAERS database.

System organ class (SOC)	Case numbers	ROR (95% CI)	PRR (χ^2^)	EBGM (EBGM05)	IC (IC025)
Vascular disorders^*^	2,171	1.15 (1.10–1.2 0)	1.15 (41.97)	1.15 (1.11)	0.20 (0.14)
Gastrointestinal disorders^*^	9,051	1.23 (1.20–1.26)	1.21 (350.47)	1.21 (1.18)	0.27 (0.24)
General disorders and administration site conditions	11,492	0.71 (0.70–0.73)	0.75 (1147.87)	0.75 (0.74)	−0.41 (-0.44)
Skin and subcutaneous tissue disorders	4,555	0.96 (0.93–0.99)	0.96 (7.12)	0.96 (0.94)	−0.06 (-0.1)
Nervous system disorders	3,798	0.49 (0.47–0.5)	0.51 (1942.03)	0.51 (0.5)	−0.97 (-1.02)
Respiratory, thoracic and mediastinal disorders^*^	7,213	1.79 (1.74–1.83)	1.72 (2288.56)	1.72 (1.69)	0.78 (0.75)
Neoplasms benign, malignant and unspecified (incl cysts and polyps)^*^	5,116	2.29 (2.22–2.35)	2.21 (3481.37)	2.21 (2.16)	1.14 (1.1)
Injury, poisoning and procedural complications	3,475	0.39 (0.38–0.41)	0.42 (3093.38)	0.42 (0.41)	−1.25 (-1.3)
Renal and urinary disorders^*^	3,933	2.51 (2.43–2.59)	2.44 (3402.08)	2.44 (2.37)	1.29 (1.24)
Blood and lymphatic system disorders^*^	9,804	7.31 (7.16–7.47)	6.60 (46900.51)	6.54 (6.43)	2.71 (2.68)
Infections and infestations^*^	5,149	1.12 (1.09–1.15)	1.11 (59.09)	1.11 (1.08)	0.15 (0.11)
Investigations^*^	6,929	1.3 (1.27–1.33)	1.28 (439.11)	1.28 (1.25)	0.35 (0.31)
Metabolism and nutrition disorders^*^	3,287	1.77 (1.71–1.84)	1.75 (1066.95)	1.74 (1.69)	0.8 (0.75)
Cardiac disorders^*^	2,544	1.1 (1.06–1.14)	1.10 (22.66)	1.10(1.06)	0.13 (0.08)
Musculoskeletal and connective tissue disorders	1,915	0.40(0.38–0.42)	0.41 (1687.1)	0.41 (0.40)	−1.27 (-1.34)
Eye disorders	799	0.45 (0.42–0.48)	0.45 (539.06)	0.45 (0.43)	−1.14 (-1.24)
Immune system disorders	696	0.71 (0.66–0.76)	0.71 (82.45)	0.71 (0.67)	−0.49 (-0.6)
Psychiatric disorders	1,054	0.2 (0.19–0.21)	0.21 (3292.29)	0.21 (0.2)	−2.24 (-2.33)
Hepatobiliary disorders^*^	2,224	2.82 (2.71–2.94)	2.78 (2540.4)	2.77 (2.67)	1.47 (1.41)
Congenital, familial and genetic disorders	170	0.63 (0.55–0.74)	0.64 (35.68)	0.64 (0.56)	−0.65 (-0.87)
Reproductive system and breast disorders	130	0.18 (0.15–0.21)	0.18 (487.24)	0.18 (0.16)	−2.47 (-2.72)
Ear and labyrinth disorders	291	0.77 (0.69–0.86)	0.77 (20.08)	0.77 (0.7)	−0.38 (-0.55)
Endocrine disorders^*^	905	4.09 (3.83–4.37)	4.06 (2078.65)	4.04 (3.82)	2.01 (1.92)
Surgical and medical procedures	297	0.25 (0.22–0.28)	0.25 (684.19)	0.25 (0.23)	−2.01 (-2.17)
Social circumstances	94	0.24 (0.2–0.3)	0.25 (218.45)	0.25 (0.21)	−2.02 (-2.32)
Product issues	27	0.02 (0.01–0.03)	0.02 (1359.22)	0.02 (0.01)	−5.68 (-6.23)
Pregnancy, puerperium and perinatal conditions	8	0.02 (0.01–0.04)	0.02 (362.1)	0.02 (0.01)	−5.55 (-6.52)

Asterisks (^*^) indicate statistically significant signals in algorithm; ROR, reporting odds ratio; PRR, proportional reporting ratio; EBGM, empirical Bayesian geometric mean; EBGM05, the lower limit of the 95% CI of EBGM; IC, information component; IC025, the lower limit of the 95% CI of the IC; CI, confidence interval; AEs, adverse events.

These findings demonstrate broad alignment with the labeled adverse events (AEs) in the pemetrexed prescribing information, with the observed associations reflecting clinically relevant signals of disproportionate reporting (SDR) for known pemetrexed toxicities. However, it is noteworthy that endocrine disorders represent a key unlabeled SDR; this signal may be confounded by underlying systemic diseases in oncology patients or concomitant medications (e.g., immune checkpoint inhibitors), and thus warrants further targeted investigations to validate potential causal associations.

### 3.3 Signals detection based on preferred term levels

**Figure 4:**
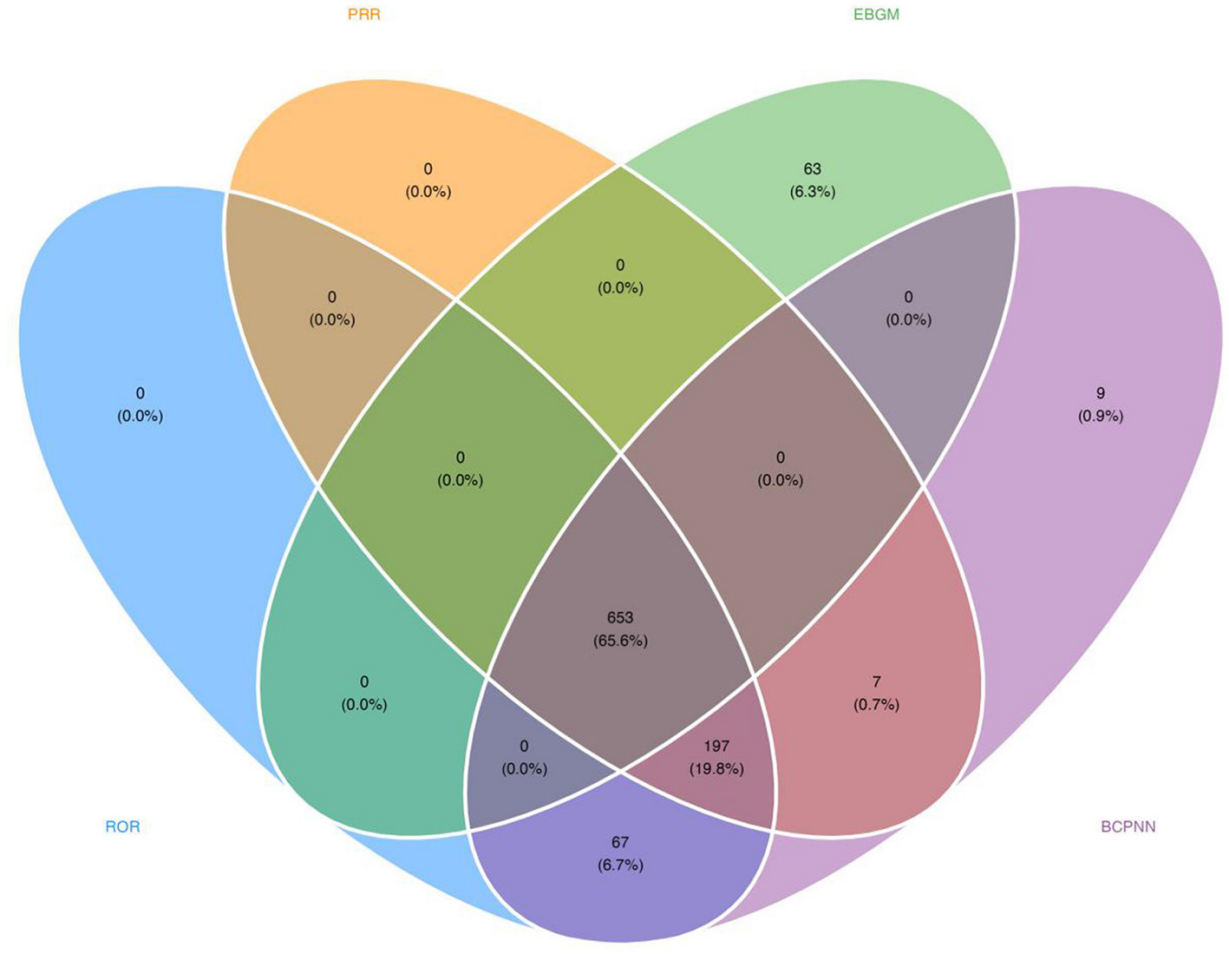
Venn diagram for the screening of all FAERS PTs based on the results of the four algorithms.

**Figure 5:**
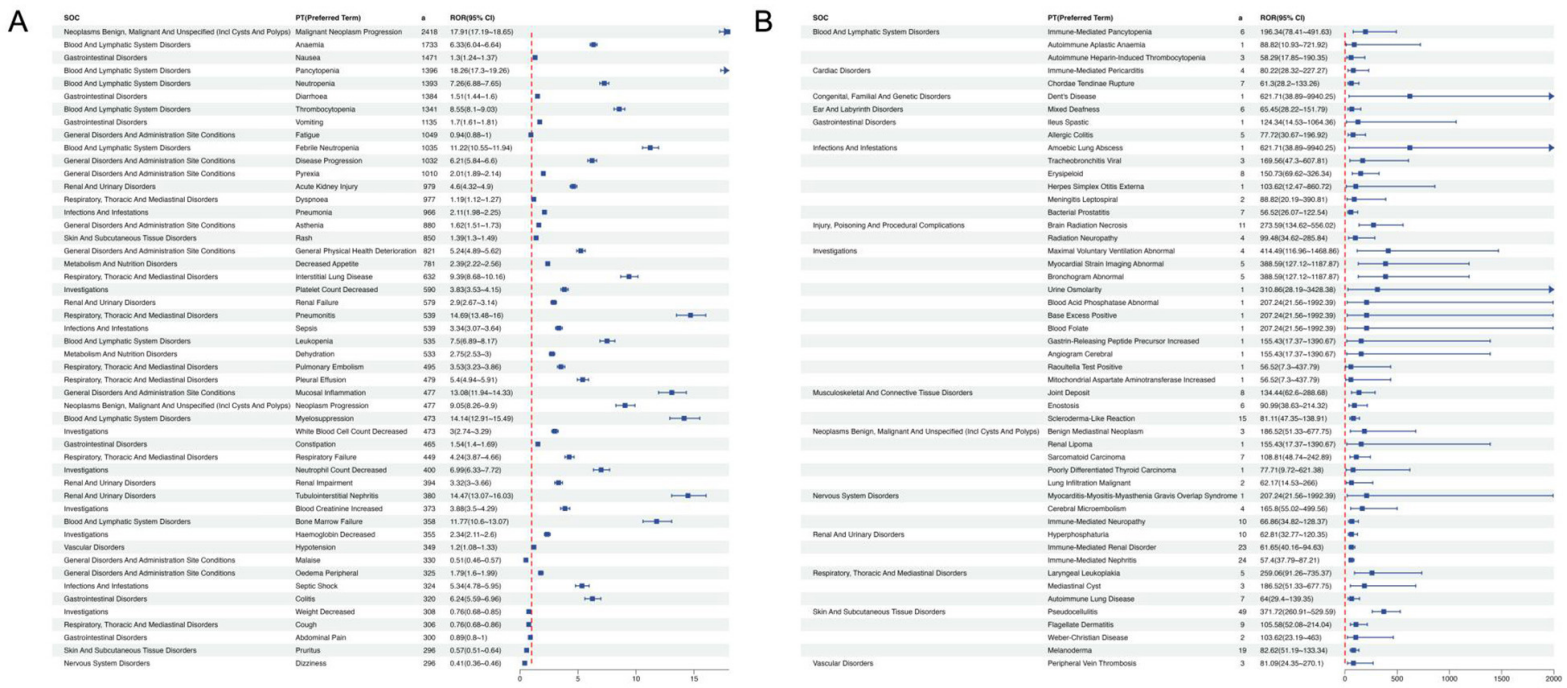
**(A)** Signal strength of reports of pemetrexed at the PT level in FAERS ranked by report count; **(B)** Signal strength of reports of pemetrexed at the PT level in FAERS ranked by ROR value.

Leveraging four disproportionality algorithms interrogating the FAERS database, 653 pemetrexed-associated positive signals of disproportionate reporting (SDR) were detected across 27 System Organ Classes (Table 3, Figure 4). Figure 5 depicts the signal identification workflow. Prioritizing PTs based on report count frequency, the top 50 events were stratified with: malignant neoplasm progression (*n* = 2,418; ROR =1 7.91, 95% CI:17.19–18.65) was the most frequent, followed by anemia (*n* = 1,733; ROR = 6.33, 95% CI:6.04–6.64), nausea (*n* = 1,471; ROR = 1.30, 95% CI: 1.24–1.37), pancytopenia (*n* = 1,396; ROR = 18.26, 95% CI: 17.30–19.26) and neutropenia (*n* = 1,393; ROR = 7.26, 95% CI: 6.88–7.65) (Figure 5A). Re-ranking SDRs by descending ROR magnitude revealed unexpectedly intense signals among low-frequency PTs, indicating significant disproportionate reporting despite limited case numbers; these low-frequency SDRs require further clinical validation to exclude confounding by underlying disease or concomitant therapies. Such as pseudocellulitis (*n* = 49; ROR = 371.72, 95% CI: 260.91–529.59), melanoderma (*n* = 19; ROR = 82.62, 95% CI: 51.19–133.34), scleroderma-like reaction (*n* = 15; ROR =8 1.11, 95% CI: 47.35–138.91), immune-mediated renal disorder (*n* = 23; ROR =6 1.65, 95% CI: 40.16–94.63), immune-mediated nephritis (*n* = 24; ROR = 57.4, 95% CI: 37.79–87.21), immune-mediated lung disease (*n* = 84; ROR = 53.39, 95% CI: 42.72–66.73), immune-mediated dermatitis (*n* = 42; ROR = 52.67, 95% CI: 38.44–72.17), hypothyroidism (*n* = 224; ROR = 4.98, 95% CI: 4.37–5.68), and hypercreatininaemia (*n* = 136; ROR = 6.69, 95% CI: 5.65–7.92) (Figure 5B).

**Table 3:** Top 50 frequency of adverse events at the PT level for pemetrexed.

PT	Case numbers	ROR (95% CI)	PRR (χ^2^)	EBGM (EBGM05)	IC (IC025)
Malignant neoplasm progression^*^	2,418	17.91 (17.19–18.65)	17.44 (36501.7)	16.99 (16.42)	4.09 (4.03)
anemia^*^	1,733	6.33 (6.04–6.64)	6.22 (7548.48)	6.17 (5.93)	2.63 (2.56)
Off label use^*^	1,708	1.44 (1.37–1.51)	1.43 (224.12)	1.43 (1.37)	0.52 (0.45)
Nausea^*^	1,471	1.30 (1.24–1.37)	1.30 (100.62)	1.30 (1.24)	0.37 (0.3)
Pancytopenia^*^	1,396	18.26 (17.3–19.26)	17.98 (21778.16)	17.5 (16.74)	4.13 (4.05)
Neutropenia^*^	1,393	7.26 (6.88–7.65)	7.16 (7310.2)	7.09 (6.78)	2.83 (2.75)
Diarrhea^*^	1,384	1.51 (1.44–1.6)	1.51 (237.06)	1.5 (1.44)	0.59 (0.51)
Thrombocytopenia^*^	1,341	8.55 (8.1–9.03)	8.43 (8683.22)	8.33 (7.96)	3.06 (2.98)
Vomiting^*^	1,135	1.7 (1.61–1.81)	1.69 (323.95)	1.69 (1.61)	0.76 (0.67)
Death	1,106	0.9 (0.84–0.95)	0.9 (13.02)	0.9 (0.85)	−0.16 (-0.24)
Fatigue	1,049	0.94 (0.88−1.00)	0.94 (4.46)	0.94 (0.89)	−0.09 (-0.18)
Febrile neutropenia^*^	1,035	11.22 (10.55–11.94)	11.10 (9356.78)	10.92 (10.37)	3.45 (3.36)
Disease progression^*^	1,032	6.21 (5.84–6.6)	6.15 (4413.46)	6.10 (5.79)	2.61 (2.52)
Pyrexia^*^	1,010	2.01 (1.89–2.14)	2.00 (502.63)	1.99 (1.89)	0.99 (0.9)
Acute kidney injury^*^	979	4.6 (4.32–4.9)	4.56 (2708.59)	4.53 (4.3)	2.18 (2.09)
Dyspnoea^*^	977	1.19 (1.12–1.27)	1.19 (30.19)	1.19 (1.13)	0.25 (0.16)
Pneumonia^*^	966	2.11 (1.98–2.25)	2.10 (554.93)	2.09 (1.98)	1.07 (0.97)
Asthenia^*^	880	1.62 (1.51–1.73)	1.61 (204.91)	1.61 (1.52)	0.69 (0.59)
Rash^*^	850	1.39 (1.3–1.49)	1.39 (92.31)	1.39 (1.31)	0.47 (0.37)
General physical health deterioration^*^	821	5.24 (4.89–5.62)	5.2 (2769.35)	5.17 (4.88)	2.37 (2.27)
Decreased appetite^*^	781	2.39 (2.22–2.56)	2.37 (621.01)	2.37 (2.23)	1.24 (1.14)
Drug ineffective	757	0.39 (0.36–0.42)	0.40 (708.11)	0.40 (0.37)	−1.33 (-1.44)
Interstitial lung disease^*^	632	9.39 (8.68–10.16)	9.33 (4632.1)	9.20 (8.62)	3.20 (3.09)
Platelet count decreased^*^	590	3.83 (3.53–4.15)	3.81 (1217.48)	3.79 (3.54)	1.92 (1.8)
Renal failure^*^	579	2.90 (2.67–3.14)	2.88 (710.15)	2.87 (2.68)	1.52 (1.4)
Sepsis^*^	539	3.34 (3.07–3.64)	3.33 (874.71)	3.32 (3.09)	1.73 (1.6)
Pneumonitis^*^	539	14.69 (13.48–16)	14.60 (6675.21)	14.29 (13.3)	3.84 (3.71)
Leukopenia^*^	535	7.50 (6.89–8.17)	7.46 (2959.9)	7.38 (6.87)	2.88 (2.76)
Dehydration^*^	533	2.75 (2.53–3)	2.74 (587.97)	2.73 (2.54)	1.45 (1.32)
Pulmonary embolism^*^	495	3.53 (3.23–3.86)	3.51 (887.11)	3.50 (3.25)	1.81 (1.68)
Pleural effusion^*^	479	5.4 (4.94–5.91)	5.38 (1693.71)	5.34 (4.95)	2.42 (2.28)
Mucosal inflammation^*^	477	13.08 (11.94–14.33)	13.02 (5184.69)	12.77 (11.83)	3.67 (3.54)
Neoplasm progression^*^	477	9.05 (8.26–9.9)	9.00 (3346.72)	8.89 (8.24)	3.15 (3.02)
White blood cell count decreased^*^	473	3.00 (2.74–3.29)	2.99 (626.26)	2.98 (2.77)	1.58 (1.44)
Myelosuppression^*^	473	14.14 (12.91–15.49)	14.07 (5617.95)	13.78 (12.77)	3.78 (3.65)
Constipation^*^	465	1.54 (1.4–1.69)	1.54 (86.97)	1.53 (1.42)	0.62 (0.48)
Respiratory failure^*^	449	4.24 (3.87–4.66)	4.23 (1099.4)	4.20 (3.89)	2.07 (1.93)
Neutrophil count decreased^*^	400	6.99 (6.33–7.72)	6.96 (2021.51)	6.90 (6.35)	2.79 (2.64)
Renal impairment^*^	394	3.32 (3–3.66)	3.31 (631.5)	3.29 (3.03)	1.72 (1.57)
Tubulointerstitial nephritis^*^	380	14.47 (13.07–16.03)	14.41 (4637.7)	14.11 (12.96)	3.82 (3.67)
Blood creatinine increased^*^	373	3.88 (3.5–4.29)	3.87 (788.38)	3.85 (3.53)	1.94 (1.79)
Bone marrow failure^*^	358	11.77 (10.6–13.07)	11.73 (3449.07)	11.53 (10.56)	3.53 (3.37)
Hemoglobin decreased^*^	355	2.34 (2.11–2.6)	2.34 (270.87)	2.33 (2.14)	1.22 (1.07)
Hypotension^*^	349	1.20 (1.08–1.33)	1.20 (11.4)	1.20 (1.1)	0.26 (0.11)
Malaise	330	0.51 (0.46–0.57)	0.51 (156.91)	0.51 (0.47)	−0.97 (-1.13)
Oedema peripheral^*^	325	1.79 (1.6–1.99)	1.78 (111.7)	1.78 (1.63)	0.83 (0.67)
Septic shock^*^	324	5.34 (4.78–5.95)	5.32 (1127.71)	5.28 (4.82)	2.4 (2.24)
Colitis^*^	320	6.24 (5.59–6.96)	6.22 (1388.01)	6.17 (5.62)	2.62 (2.46)
Weight decreased	308	0.76 (0.68–0.85)	0.76 (23.48)	0.76 (0.69)	−0.40 (-0.56)
Cough	306	0.76 (0.68–0.86)	0.77 (21.99)	0.77 (0.7)	−0.38 (-0.55)

Asterisks (^*^) indicate statistically significant signals in algorithm; ROR, reporting odds ratio; PRR, proportional reporting ratio; EBGM, empirical Bayesian geometric mean; EBGM05, the lower limit of the 95% CI of EBGM; IC, information component; IC025, the lower limit of the 95% CI of the IC; CI, confidence interval; PT, preferred term.

### 3.4 Subgroup analysis

**Figure 6:**
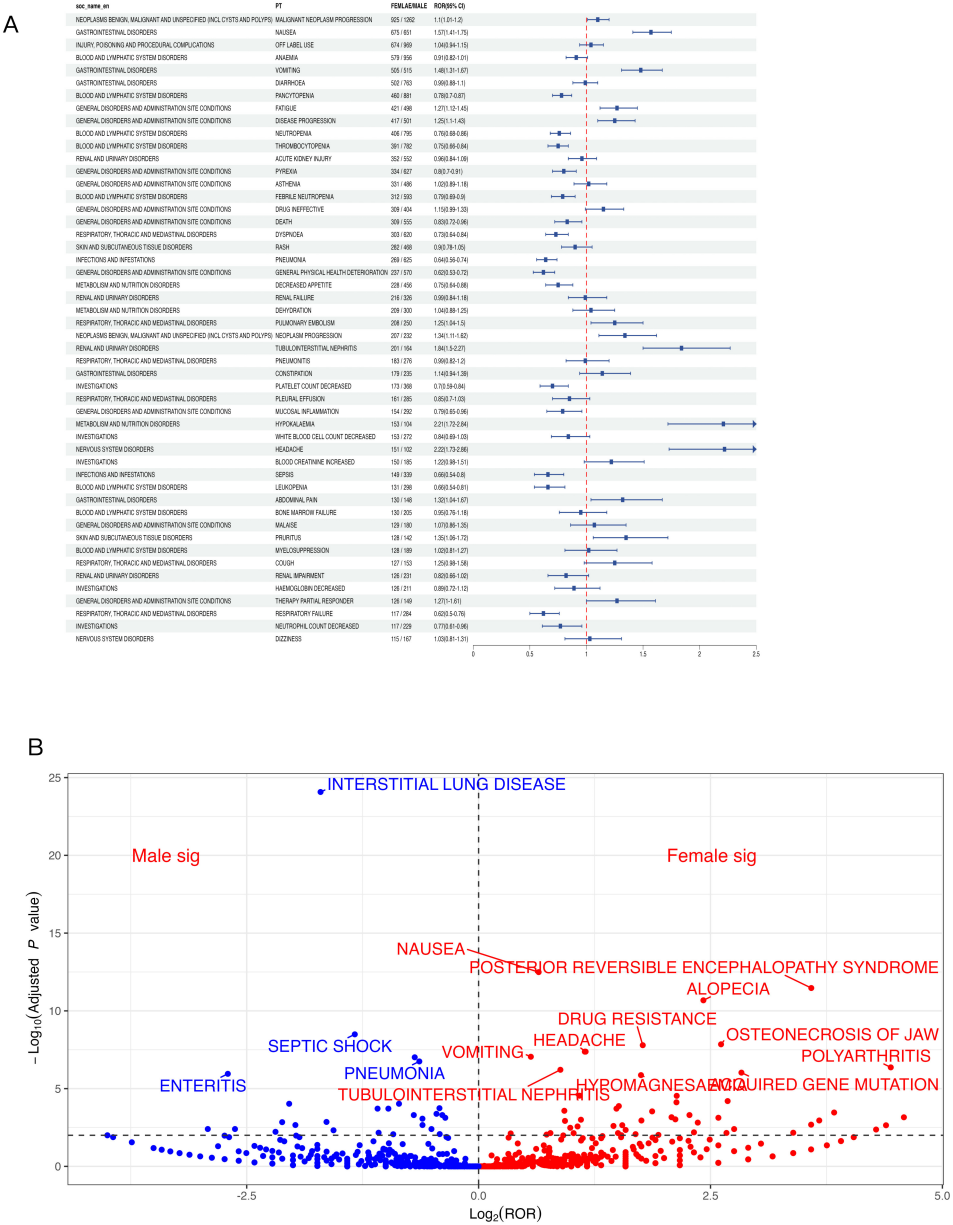
Subgroup analysis of pemetrexed-associated safety signals by gender. **(A)** Overview of the top 50 most frequent events in male and female patients. **(B)** Top 25 preferred terms in male and female subgroups. Disproportionality analysis based on reporting odds ratios. For improved clarity, detailed supporting data are provided in Supplementary Figure S6.

Subgroup analysis of pemetrexed-related signals of disproportionate reporting (SDRs) to explore risk patterns across different sexes, age groups, and reporter types. Sex subgroup analysis identified differences in the reporting of pemetrexed-associated SDRs. The top 50 most frequently reported AEs in Figure 6A. Figure 6B delineates the top 25 most frequently reported preferred terms (PTs) of adverse reactions in the male and female cohorts. Supplementary Figure S6 presents sex-specific disproportionality signals based on ROR values, highlighting distinct strong positive safety signals in male and female cohorts.

The results show that certain AEs were more frequently reported in male patients, particularly those related to respiratory, thoracic and mediastinal disorders. These included interstitial lung disease (ROR = 8.96, 95% CI: 8.16–9.83) and pneumonitis (ROR = 11.58, 95% CI: 10.27–13.05). Additional PTs with strong positive signals included septic shock and enteritis. However, female patients demonstrated a higher predisposition to renal and urinary adverse events, exemplified by tubulointerstitial Nephritis (ROR = 30.94, 95% CI: 26.87–35.62). Additional strong positive signals in female patients included: nausea, posterior reversible encephalopathy syndrome, alopecia, drug resistance, headache, vomiting, osteonecrosis of the jaw, polyarthritis, hypomagnesaemia, and acquired gene mutation (Supplementary Tables S4, S5).

**Figure 7:**
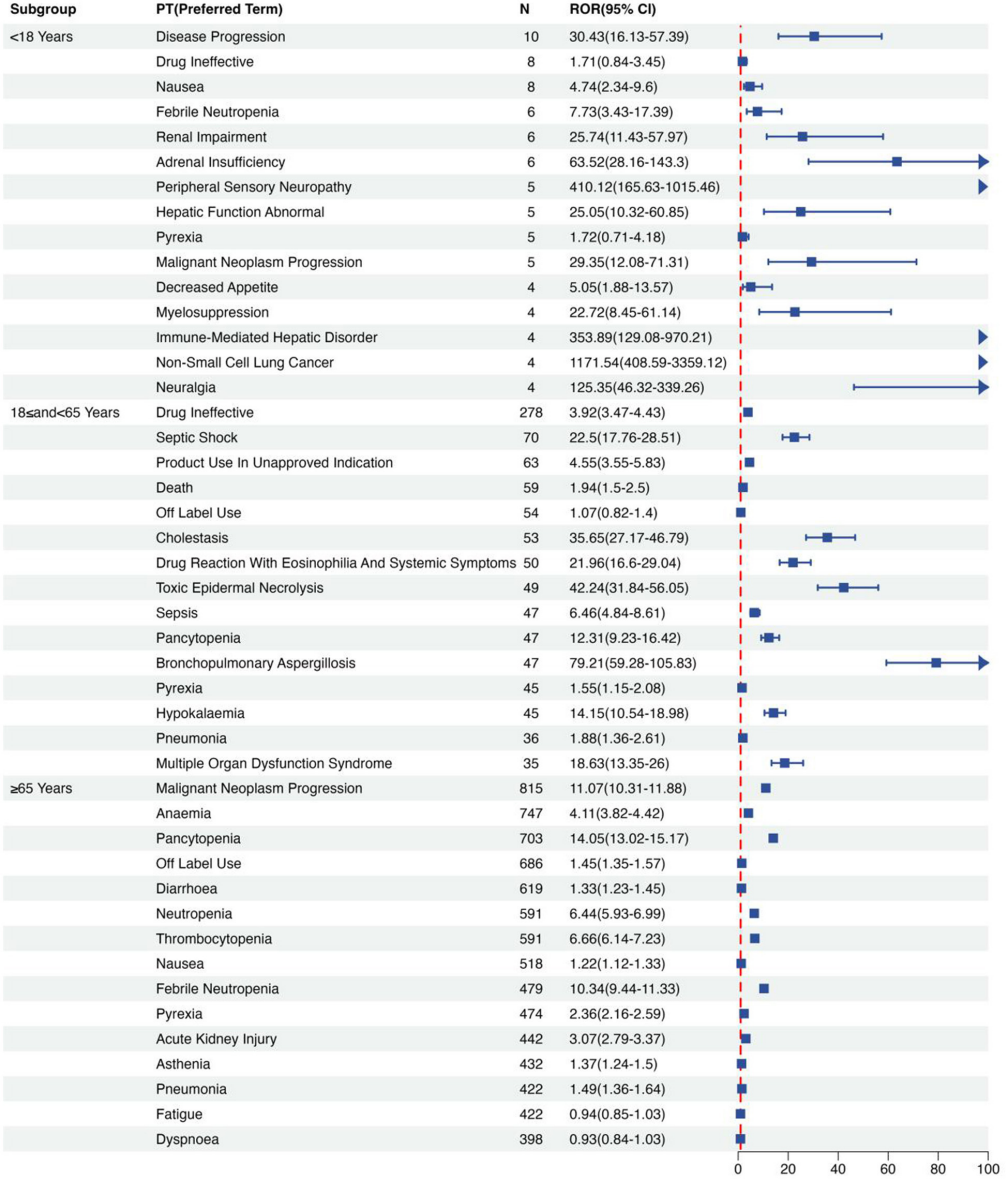
Signal strength for pemetrexed-associated AEs in age subgroup.

Age subgroup analysis identified differences in the reporting of pemetrexed-associated SDRs between the pediatric cohort (< 18 years), adult cohort (18– < 65 years) and geriatric cohort (≥65 years). The top 15 most frequently reported SDRs in Figure 7. The pediatric cohort demonstrated extreme disproportionality for neurological and metabolic toxicity-related SDRs, including peripheral sensory neuropathy (ROR = 410.12, 95% CI: 165.63–1015.46), immune-mediated hepatic disorder (ROR = 353.89, 95% CI: 129.08–970.21), and adrenal insufficiency (ROR = 63.52, 95% CI: 28.16–143.30). Notably, the non-small cell lung cancer indication showed anomalous reporting (ROR = 1171.54, 95% CI: 408.59–3359.12), potentially reflecting off-label use documentation errors. Immunoallergic and infection complication-related SDRs dominated the adult cohort, such as toxic epidermal necrolysis (ROR = 42.24, 95% CI: 31.84–56.05), bronchopulmonary aspergillosis (ROR = 79.21, 95% CI: 59.28–105.83), and drug reaction with eosinophilia and systemic symptoms (DRESS, ROR = 21.96, 95% CI: 16.60–29.04). Among the geriatric cohort, hematologic and organ dysfunction-related SDRs predominated: pancytopenia (ROR = 14.05, 95% CI: 13.02–15.17), febrile neutropenia (ROR = 10.34, 95% CI: 9.44–11.33) and multiple organ dysfunction syndrome (ROR = 18.63, 95% CI: 13.35–26.00). Unexpectedly, malignant neoplasm progression exhibited strong signal strength (ROR = 11.07, 95% CI: 10.31–11.88), suggesting possible confounding by indication (Supplementary Tables S6, S8).

**Figure 8:**
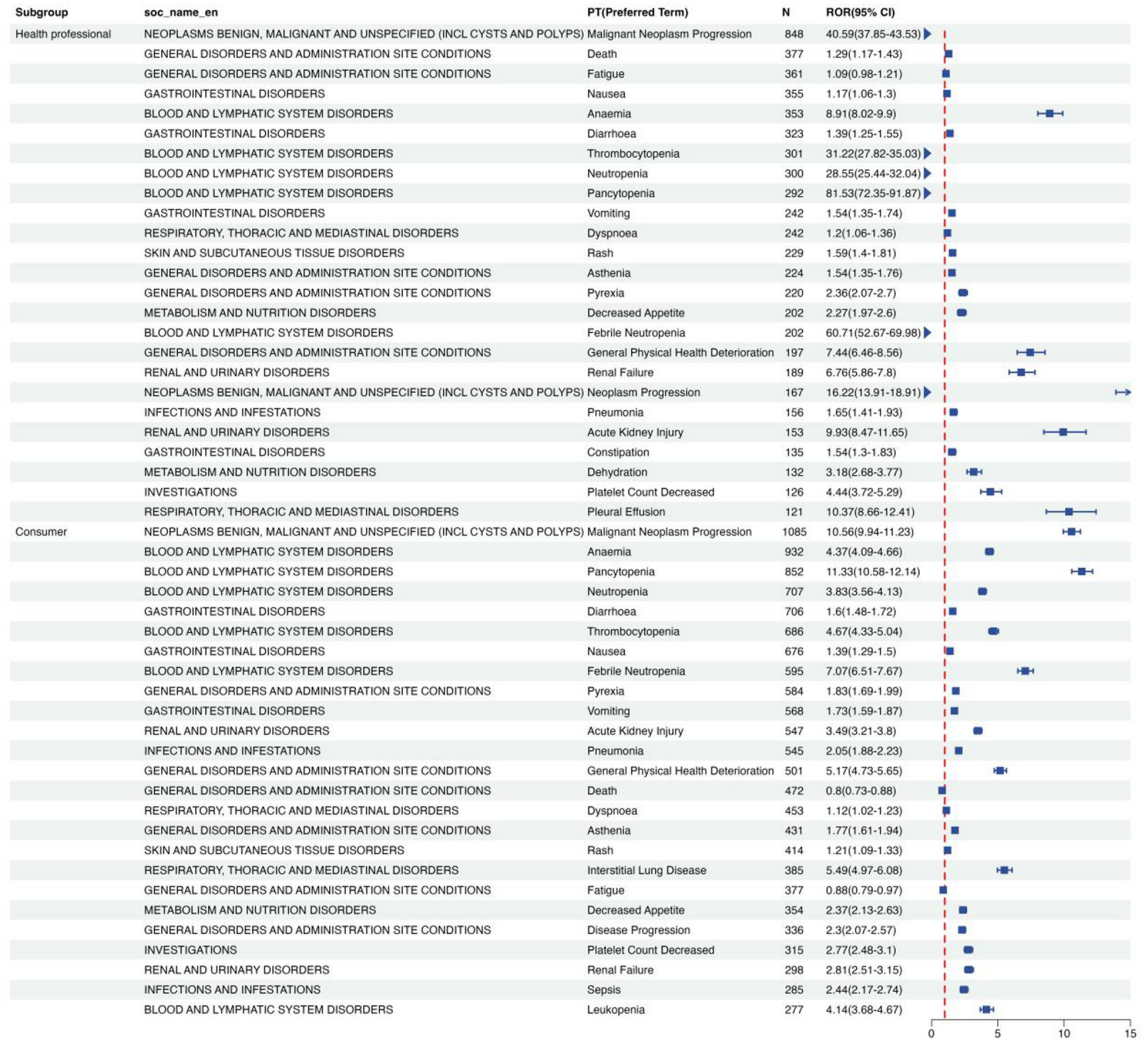
Signal strength for pemetrexed-associated AEs in reports subgroup.

Quantitative signal detection identified significant reporter-related discrepancies in pemetrexed-associated SDR profiles. Healthcare professionals (HCPs) submitted 10,073 reports, while consumers contributed 6,074 reports. HCPs demonstrated heightened reporting of hematologic, and malignant progression-related SDRs: pancytopenia (ROR = 81.53; 95% CI: 72.35–91.87), febrile neutropenia (ROR = 60.71; 95% CI: 52.67–69.98), malignant neoplasm progression (ROR = 40.59; 95% CI: 37.85–43.53), thrombocytopenia (ROR = 31.22; 95% CI: 27.82–35.03), and neutropenia (ROR = 28.55; 95% CI: 25.44–32.04). Notably, renal and urinary disorders demonstrated increased reporting disproportionality signals at both SOC and PT levels: acute kidney injury (ROR = 9.93; 95% CI: 8.47–11.65) and renal failure (ROR = 6.76; 95% CI: 5.86–7.80). However, consumers emphasized symptomatic and quality-of-life impact-related SDRs, such as anemia (*n* = 932; ROR = 4.37; 95% CI: 4.09–4.66), diarrhea (ROR = 1.60), nausea (ROR = 1.39), and vomiting (ROR = 1.73). A substantial proportion of reports were coded with serious outcomes (including death) among pemetrexed-associated reports in FAERS. Death is routinely documented and reported as a serious outcome by HCPs due to professional reporting obligations, while consumer reports rarely document death outcomes due to their focus on subjective symptomatic discomfort and lack of formal reporting mandates. Death reports in the consumer subgroup showed an inverse association (ROR = 0.80; 95% CI: 0.73–0.88), contrasting with HCPs' positive signal (ROR = 1.29; 95% CI: 1.17–1.43) (Figure 8). This inverse association of death in consumer reports reflects differential reporting patterns between the two groups and does not indicate a reduced pemetrexed-related mortality risk in the population corresponding to consumer reports. Collectively, disproportionality metrics from FAERS reflect reporting patterns rather than actual clinical incidence or causal associations, and all identified SDRs should be interpreted as hypothesis-generating findings for subsequent clinical validation. To characterize disparities in SDR patterns across subgroups, we computed subgroup-specific ROR values with 95% CIs and assessed the overlap of confidence intervals to infer potential differences. Formal statistical tests for interaction (e.g., sex × SDR occurrence, age × SDR occurrence) were not performed, given the observational nature of FAERS data and residual confounding from unrecorded factors (e.g., comorbidities, prior therapies). Thus, the observed subgroup differences are descriptive and require validation in hypothesis-driven studies with controlled designs.

### 3.5 Time-to-onset analysis

**Figure 9:**
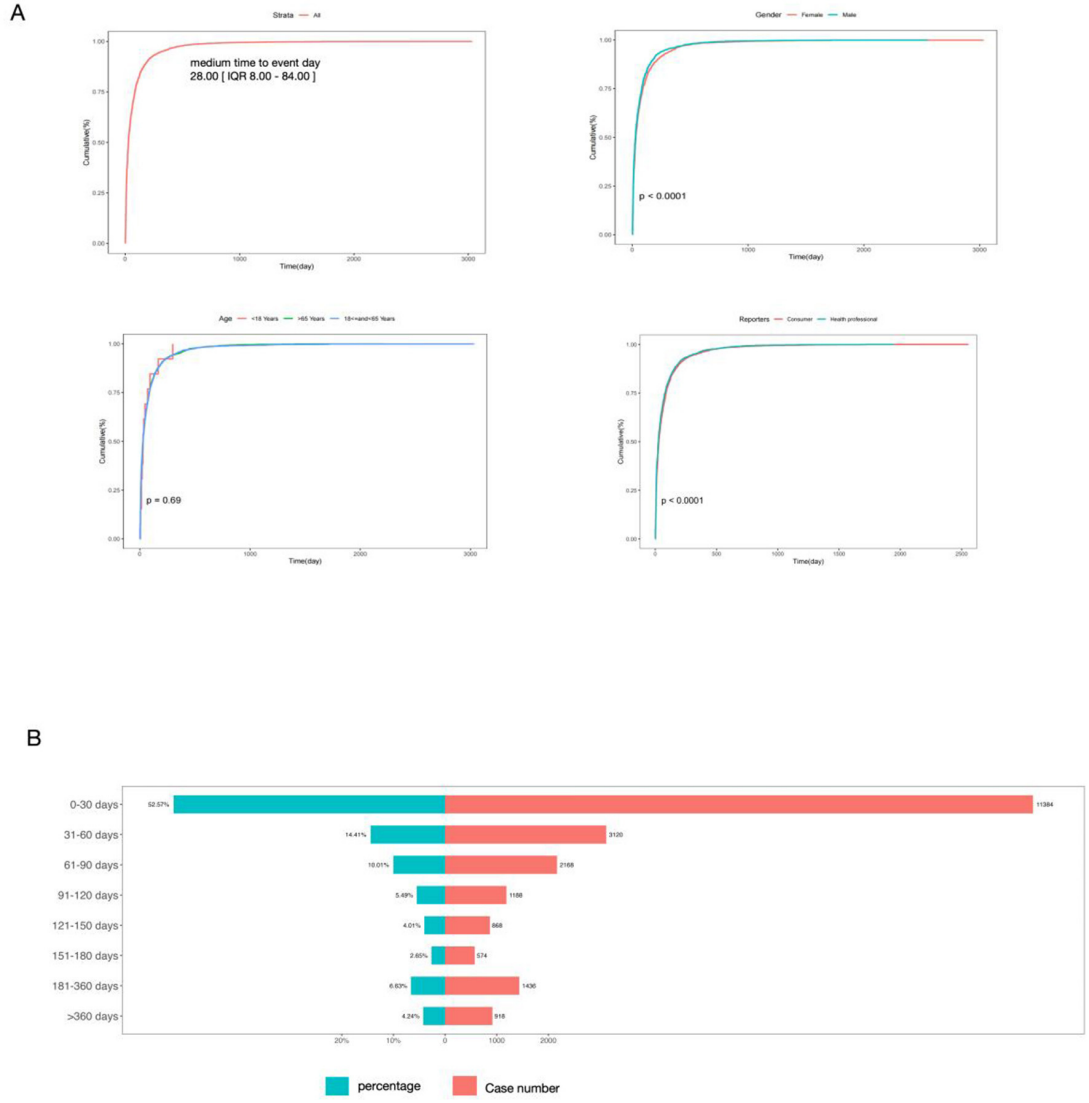
Time-to-onset (TTO) analysis of patients treated with pemetrexed. **(A)** Cumulative incidence of patients stratified by age, sex, and reporters. **(B)** Number and proportion of patients stratified by time-to-onset.

Analysis of 21,658 adverse event reports (signals) revealed a bimodal onset distribution: 52.57% (*n* =1 1,384) occurred within 0–30 days post-treatment, 14.41% (*n* = 3,121) during 31–60 days, and secondary peak at 181–360 days (6.63%, *n* = 1,436) (Figure 9A). The cumulative incidence of time-to-onset is depicted in Figure 9B. Significant divergence in incidence trajectories by sex and report type was observed (*P* < 0.0001, Figure 9A). In contrast, age stratification demonstrated no statistically significant differences across predefined strata (*P* = 0.69, Figure 9B). Additionally, Weibull distribution analysis was performed to quantitatively characterize the time-to-onset pattern, with detailed parameters listed in Table 4. The two core parameters of the Weibull distribution are interpreted as follows: (1) Scale parameter (α = 57.99, 95% CI = 56.80–59.18): Also referred to as “characteristic life,” it represents the time point when the cumulative probability of SDR onset reaches ~63.2% (1–1/e) in the overall population—indicating ~63.2% of pemetrexed-associated SDRs occurred within 58 days after treatment initiation. (2) Shape parameter (β = 0.69, 95% CI = 0.68–0.69): Determines the trend of onset risk over time; a β value < 1 indicates an “early failure mode” (decreasing failure rate), meaning the risk of SDR onset is highest in the early stage post-administration and gradually decreases over time—consistent with the prominent early peak of the bimodal pattern.

**Table 4:** Weibull distribution parameters for time-to-onset of pemetrexed-related SDRs.

Drug	TTO (days)		Weibull distribution		
	Case reports	Median (d) (IQR)	Scale parameter: α (95% CI)	Shape parameter: β (95% CI)	Type
Pemetrexed	21,656	28 (8–84)	57.99 (56.80–59.18)	0.69 (0.68–0.69)	Early failure

TTO, time-to-onset; CI, confidence interval; IQR, interquartile range.

Time-to-onset (TTO) was calculated as EVENT_DT – START_DT, with median and interquartile range (IQR) reported for descriptive summary.

### 3.6 Sensitivity analysis

Considering that pemetrexed is often used in clinical combination therapy, and secondary suspect (SS)/concomitant drugs may introduce confounding bias even when pemetrexed is the unique PS, we excluded reports involving common co-administered drugs (Cisplatin, carboplatin, pembrolizumab, bevacizumab). After excluding these four drugs from the 27,098 valid PS reports, 1,951 reports remained, of which 863 contained significant AE signals.

The top 10 significant SDRs with the highest ROR values in this non-combination therapy subset included abulia (ROR = 12.35, 95% CI = 8.62–17.68), hypoxia (ROR = 9.87, 95% CI = 7.12–13.65), erythema (ROR = 8.92, 95% CI = 6.58–12.09), pyrexia (ROR = 7.63, 95% CI = 6.15–9.45), peripheral oedema (ROR = 6.89, 95% CI = 5.56–8.52), anemia (ROR = 6.31, 95% CI = 5.28–7.54), dyspnoea (ROR = 5.92, 95% CI = 4.89–7.17), fatigue (ROR = 5.16, 95% CI = 4.23–6.29), nausea (ROR = 4.83, 95% CI = 4.01–5.82), and thrombocytopenia (ROR = 4.57, 95% CI = 3.79–5.49). Notably, the core safety SDRs of pemetrexed (e.g., haematologic toxicity, general systemic symptoms, and mild cutaneous reactions) remained statistically significant in this non-combination subset, consistent with the findings of the full dataset. The complete statistical results of all positive SDRs in the sensitivity analysis, including case numbers, ROR, PRR, EBGM, and IC values, are detailed in Supplementary Table S9.

## 4 Discussion

### 4.1 Baseline characteristic analysis of pemetrexed-associated ADEs

This study included 27,098 pemetrexed-associated reports from FAERS (pemetrexed as unique PS), identifying 653 signals of disproportionate reporting (SDRs) across 27 SOCs; these SDRs reflect temporal associations with pemetrexed exposure but not confirmed causal AEs, consistent with spontaneous reporting system limitations. Reports among adults aged ≥18 years showed comparable frequencies, aligning with prescribing information indicating no significant increase in adverse event risk for patients aged ≥65 vs. < 65 years in clinical trials. Male reports predominated (54.80%) over females (35.60%), likely attributable to pemetrexed's primary indication for lung malignancy, wherein male incidence rates approximate double those of females (10). The top two indications were malignant lung neoplasms and mesothelioma, aligning with clinical applications and FDA-approved indications. Over half of the reports were submitted by physicians and healthcare professionals, supporting the reliability of our findings (11, 12). Reports spanned approximately 72 countries/regions, with the United States submitting the highest volume—consistent with FAERS' origin as a US-established database.

### 4.2 Analysis of SOC involvement in pemetrexed-associated adverse events

This study employed a pharmacovigilance framework by integrating four disproportionality approaches (ROR, PRR, EBGM, and BCPNN) to detect and prioritize pemetrexed-associated AE signals. This framework revealed a broad safety-signal profile across multiple organ systems; however, selected SDR signals (e.g., neoplasm-related terms) may represent disease progression or lack of efficacy rather than adverse reactions. Consequently, comprehensive clinical monitoring and prompt interventions are warranted during pemetrexed therapy.

Beyond enumerating signal frequencies, it is clinically more informative to interpret these SOC patterns in the context of pemetrexed's pharmacology and the underlying oncology population. Several high-volume SOCs (e.g., general disorders, hematologic and gastrointestinal events) likely reflect a composite of treatment-related toxicity, supportive care-associated reporting, and cancer-related symptomatology. In contrast, a subset of disproportionality signals (e.g., immune-mediated endocrine, renal, dermatologic, and pulmonary manifestations) constitutes hypothesis-generating safety cues requiring targeted clinical vigilance and mechanistic validation. Accordingly, we focus the discussion below on clinical plausibility, potential mechanisms, and actionable monitoring implications rather than restating percentages.

At the SOC level, signals clustered mainly in systemic symptoms, hematologic and gastrointestinal toxicities, infections, renal events, and respiratory conditions (Table 2; Supplementary Tables S4–S9). Importantly, FAERS is a spontaneous reporting system in which events are recorded in temporal association with drug exposure, and causal attribution is inherently limited. Patients with lung cancer and mesothelioma frequently receive multiple prior and/or concomitant systemic therapies (e.g., platinum agents, radiotherapy, and immune checkpoint inhibitors). Therefore, some reported events—particularly those with overlapping toxicity profiles or delayed immune-related manifestations—may not be directly attributable to pemetrexed alone. In addition, signals captured under the SOC *Neoplasms benign, malignant, and unspecified* may reflect disease progression or lack of therapeutic efficacy (i.e., treatment failure/drug resistance) rather than true adverse reactions. Accordingly, these findings should be interpreted cautiously with respect to causality, and further prospective or well-controlled studies are warranted to validate the observed signals.

Critically, we also detected a previously unlabeled SDR for endocrine disorders (27 PTs, 129 cases, 1.04%) including hypothyroidism, hyperthyroidism, and adrenal insufficiency. This endocrine SDR represents a potential novel safety finding not described in current labeling, but may be confounded by multiple factors inherent to oncology populations: (1) underlying systemic malignancy and paraneoplastic endocrine dysfunction; (2) concomitant administration of immune checkpoint inhibitors, which are well-documented to induce immune-mediated endocrine toxicities; (3) cumulative organ damage from prior anticancer therapies. Accordingly, this SDR is hypothesis-generating and requires prospective controlled studies to evaluate potential causal links with pemetrexed exposure.

Notably, General disorders and administration site conditions accounted for the largest case volume (13.19%). This SOC in oncology pharmacovigilance often aggregates non-specific but clinically consequential symptom clusters (e.g., pyrexia, asthenia/fatigue, edema, and generalized deterioration) that may arise from chemotherapy-related systemic inflammation, infusion/administration effects, or intercurrent complications such as infection and febrile neutropenia. Because these presentations are highly confounded by the underlying malignancy and concomitant therapies, we interpret them primarily as signals for clinical prioritization (prompt infection workup, early recognition of febrile neutropenia, hydration/supportive care, and patient education on red-flag symptoms) rather than as drug-specific causal endpoints.

### 4.3 Blood and lymphatic system disorders

The principal toxicity identified during the clinical development of pemetrexed was myelosuppression, with 50% of patients experiencing grade 3/4 neutropenia (13). Our study found that blood and lymphatic system disorders constituted the most frequently reported SOC, with high-incidence PTs including anemia, pancytopenia, neutropenia, and thrombocytopenia. Myelosuppression, notably neutropenia, is the primary dose-limiting toxicity, which may necessitate treatment delays/discontinuation, hospitalization, or even death (14). In a phase II NSCLC trial of pemetrexed monotherapy (600 mg/m^2^) without vitamin supplementation, 41% of patients developed grade 3/4 neutropenia (15). Consequently, implementing targeted prophylaxis against hematologic toxicity is critical. Current prescribing guidelines advise pemetrexed-treated patients to initiate oral folic acid supplementation (400–1,000 μg daily) starting 7 days before the first dose and continuing until 21 days after the final dose. Concurrently, an initial intramuscular vitamin B_12_ injection (1,000 μg) should be administered during the week preceding cycle one, followed by maintenance doses every three treatment cycles, subsequent B_12_ injections may be administered on pemetrexed dosing days. With folic acid and vitamin B12 supplementation, the frequency of severe toxicity was substantially lower; for example, the rate of grade 4 hematologic or grade 3/4 non-hematologic toxicity decreased from 37% in unsupplemented patients to 6.4% in supplemented patients in analyses from early pemetrexed programs (16, 17). Evidence from studies (18–20) indicates that initiating folic acid and vitamin B_12_ supplementation 1 week prior to pemetrexed therapy yields no significant reduction in hematologic toxicity. However, shortened vitamin lead-in (≈24 h before pemetrexed initiation) has been evaluated in a prospective study and was generally well-tolerated, suggesting feasibility for earlier chemotherapy initiation; however, evidence remains limited and routine practice should follow product labeling and local guidelines (18).

#### 4.3.1 Gastrointestinal disorders

Pemetrexed frequently causes gastrointestinal adverse reactions such as nausea, vomiting, diarrhea, anorexia, and mucosal inflammation. Cisplatin combination therapy demonstrably elevates their incidence (21). Our study detected significant gastrointestinal immune-mediated signals of disproportionate reporting (SDRs) such as colitis, esophagitis, oral papule, immune-mediated pancreatitis, immune-mediated enterocolitis, and autoimmune colitis-all currently unlisted in drug labeling. Notably, these gastrointestinal immune-mediated SDRs may be confounded by prior or concurrent use of immune checkpoint inhibitors (ICIs), as ICIs are well-recognized to induce immune-related adverse events (irAEs) involving the gastrointestinal tract (e.g., immune-mediated colitis) with clinical manifestations consistent with the SDRs identified in this study. Our sensitivity analysis excluded pembrolizumab-containing reports, and colitis remained a significant SDR in this non-ICI subset—suggesting a potential association with pemetrexed that requires further validation. Grade 3/4 nausea and vomiting occur in 9–14% of patients without vitamin supplementation, but are effectively mitigated by prophylactic vitamins. For pemetrexed-treated patients, strict adherence to folic acid/vitamin B12 protocols and preemptive antiemetics are essential. A low-residue, non-irritating diets and meticulous oral hygiene prevent mucositis and oral papules. Proactive antidiarrheal therapy with continuous surveillance is critical—severe immune-mediated manifestations (autoimmune colitis, immune-mediated pancreatitis/enterocolitis) require immediate treatment discontinuation and corticosteroid support.

#### 4.3.2 Respiratory, thoracic, and mediastinal disorders

Understanding these events is critical for optimizing clinical management and improving patient safety. Post-marketing surveillance has documented respiratory and mediastinal adverse reactions to pemetrexed, including interstitial pneumonitis and radiation recall pneumonitis. Direct cytotoxic damage to alveolar epithelial cells triggers pro-inflammatory cytokine release (IL-6, TNF-α), leading to fibroblast activation and fibrosis (22). This is exacerbated by impaired DNA synthesis due to thymidylate synthase inhibition. Our research identified significant disproportionality signals within the respiratory, thoracic and mediastinal disorders, including pneumonitis, immune-mediated lung disease, hypersensitivity pneumonitis, pulmonary toxicity, and lung consolidation. Notably, these PTs exhibiting the strongest safety signals are not listed in the current drug labeling. These respiratory immune-mediated SDRs may be affected by the confounding of prior/concurrent ICI use, as ICIs are a well-documented cause of immune-mediated pneumonitis—a severe irAE with clinical features overlapping those of the pemetrexed-associated SDRs identified in this study. Although pembrolizumab was excluded in the sensitivity analysis, the immune-mediated lung disease SDR remained significant in the non-combination subset-this does not rule out an association with pemetrexed itself or the confounding of other unexcluded ICIs (e.g., nivolumab). A 2019 case report documented grade 4 anaphylaxis during a later pemetrexed cycle despite prior tolerance. Though a hypersensitivity reaction, anaphylaxis is clinically relevant to respiratory toxicities due to dominant acute respiratory compromise (e.g., airway edema, bronchospasm, severe dyspnea). Beyond pneumonitis and other pulmonary toxicities, clinicians must remain alert to acute hypersensitivity with respiratory involvement and manage it promptly per standard emergency protocols. Labeled and institutional premedication regimens aim to reduce hypersensitivity and cutaneous reactions, but evidence for preventing rare severe anaphylaxis is limited—rapid recognition and treatment preparedness are essential (23). In cases of severe interstitial pneumonitis, permanent discontinuation of pemetrexed is mandated. Additional clinical research is needed to develop safer interventions for pemetrexed-related respiratory AEs.

#### 4.3.3 Renal and urinary disorders

Renal and urinary disorders constitute a critical SOC for pemetrexed-associated SDRs. In our study, frequently reported PTs within this SOC included renal failure, acute kidney injury, renal impairment, and tubulointerstitial nephritis-the latter representing an unlabeled SDR not documented in current prescribing information. This tubulointerstitial nephritis SDR may be confounded by concomitant nephrotoxic medications (e.g., platinum agents) or underlying renal dysfunction related to the primary malignancy, and thus requires further validation. Strong disproportionality signals were observed for hyperphosphaturia, immune-mediated nephritis, autoimmune nephritis, immune-mediated cystitis, and renal arteriosclerosis-all unlisted in drug labeling. These renal immune-mediated SDRs have potential confounding from prior/concurrent ICI use, as ICIs can induce immune-mediated nephritis and tubulointerstitial nephritis-consistent with the unlabeled SDRs identified in this study. Our sensitivity analysis excluded pembrolizumab-related reports, and tubulointerstitial nephritis remained significant in this non-ICI subset—indicating the signal may not be solely ICI-related and warranting further exploration of its association with pemetrexed.

Evidence indicates that prolonged pemetrexed therapy significantly elevates renal toxicity risk, suggesting cumulative dosing as a key risk determinant (24). Furthermore, nephrotoxicity may be the primary dose-limiting factor for long-term pemetrexed use. Per prescribing guidelines, treatment must be discontinued if creatinine clearance falls below 45 mL/min. Impaired renal function increases systemic pemetrexed exposure, potentiating other toxicities. Notably, renal toxicity co-occurred with hematologic toxicity in approximately one-third of patients (20). Renal-related disproportionality signals require cautious interpretation, as FAERS lacks incidence data and causal confirmation. Nevertheless, these signals are clinically plausible in oncology contexts and align with existing recommendations for renal function monitoring during pemetrexed therapy—particularly in patients with baseline renal impairment or concomitant nephrotoxic exposures. Our findings support heightened clinical awareness and appropriate renal monitoring as part of routine safety surveillance, without implying causality.

#### 4.3.4 Infections and infestations

Infections/infestations represent a critical SOC for pemetrexed-associated AEs (55 PTs). Common PTs include sepsis, septic shock, neutropenic sepsis, erysipelas, and peritonitis; unlabeled PTs with strong disproportionality signals include pneumothorax, bacterial pleural infection, dermatitis/subcutaneous inflammation, and visceral leishmaniasis. As an antimetabolite, pemetrexed induces hematotoxicity leading to myelosuppression and subsequent immune impairment, which elevates infection risk. Clinical recommendations include: (1) routine monitoring of hematologic parameters and infection biomarkers; (2) prompt initiation of targeted antibiotics for documented infections; (3) granulocyte colony-stimulating factor (G-CSF) prophylaxis or treatment for severe neutropenia.

#### 4.3.5 Skin and subcutaneous tissue disorders

Our study identified 40 skin and subcutaneous tissue disorder PTs: labeled common PTs (maculopapular rash, Stevens-Johnson syndrome, rash, and drug eruption); unlabeled common PTs (purpura and bullous dermatitis); strong-signal unlabeled PTs (pseudocellulitis, flagellate, melanoderma, immune-mediated, and autoimmune dermatitis). The potential mechanism involves pemetrexed-induced myelosuppression (reduced leukocyte and platelet counts), which triggers cutaneous inflammation or purpura. Guidelines recommend dexamethasone (4 mg twice daily, pre-infusion, intra-infusion, and post-infusion) for mitigating cutaneous AEs, however dosing is controversial—single 20 mg doses have been proposed to improve patient compliance.

#### 4.3.6 ADEs of other SOC

Pemetrexed-associated AEs involve multiple organ systems with quantitative disproportionality analysis identifying unrecognized signals. In the neurological disorders SOC, polyneuropathy and peripheral sensory neuropathy are labeled reactions, whereas cerebral microembolism, immune-mediated neuropathy, and myasthenic syndrome represent unlabeled signals. Pharmacodynamic evidence suggests that pemetrexed may cause neurotoxicity by impairing folate metabolism, inhibiting nucleotide synthesis, compromising DNA repair, and disrupting axonal function. In the metabolism and nutrition disorders SOC, decreased appetite, dehydration, and electrolyte disturbances are labeled; however, hypercreatininemia and hyperamylasemia are unlabeled signals potentially related to nephrotoxicity and pancreatotoxicity, consistent with immune-mediated pancreatitis. The underlying pathogenesis remains incompletely understood but likely involves inflammatory cascades leading to pancreatic dysfunction and amylase hypersecretion (25). Clinically, dehydration secondary to severe vomiting or diarrhea may potentiate renal impairment, significantly increasing the risk of acute kidney injury (AKI) in patients with preexisting hypercreatininemia. Our study identified potential adverse reactions within the Cardiac Disorders SOC, including immune-mediated pericarditis, autoimmune myocarditis, pericardial effusion, acute coronary syndrome and cardiac insufficiency—all currently unlisted in the prescribing information. Proposed pathophysiological processes implicate direct mitochondrial cardiotoxicity and oxidative-inflammatory cascade responses (26). Given these cardiac risks, vigilant monitoring and prompt management of cardiovascular complications post-pemetrexed administration are critical. For hepatobiliary disorders, hepatic dysfunction is a labeled, frequently reported reaction; however, hepatitis, cholestasis, cholangitis, and autoimmune hepatitis—potential pemetrexed-related events—remain unlabeled. Pemetrexed-associated liver injury is relatively common, manageable, and may correlate with favorable treatment response (27).

For patients with hepatic impairment or underlying liver disease, close liver enzyme monitoring is essential to mitigate severe adverse event risk. Disproportionality analysis identified novel pemetrexed-associated endocrine abnormalities (hypothyroidism, hyperthyroidism, adrenal insufficiency, hypopituitarism, thyroiditis)—not documented in prescribing information. Mechanisms remain unclear but may involve immune-mediated thyroid injury and endocrine dysregulation, disrupting thyroid hormone synthesis and hypothalamic-pituitary-thyroid axis function. Endocrine signals require cautious interpretation: accounting for ~1% of reports, FAERS lacks baseline endocrine data, laboratory findings, and longitudinal follow-up, precluding distinction between treatment-related effects, comorbidities, or disease-associated metabolic changes. These hypothesis-generating findings support clinical awareness and consideration of endocrine evaluation in symptomatic or long-term treated patients, not universal routine monitoring.

In summary, SOC event frequencies in FAERS reflect reporting patterns and triage priorities, rather than true incidence. All results require clinical context for interpretation and validation via well-controlled studies.

#### 4.3.7 Implications of sensitivity analysis for combination therapy

A sensitivity analysis excluding the four most common combined drugs (cisplatin, carboplatin, pembrolizumab, bevacizumab) was performed to eliminate confounding from drug-drug interactions on pemetrexed-associated SDRs, and the results confirmed the robustness of pemetrexed's core safety signals identified in the full dataset. First, core SDRs related to pemetrexed's known toxicities (e.g., anemia, thrombocytopenia, pyrexia) remained statistically significant in the non-combination subset, eliminating confounders from platinum-based drugs (nephrotoxic, myelosuppressive) and immune checkpoint inhibitors (immune-mediated toxicities). This reinforces a reporting-level association between these core signals and pemetrexed exposure, rather than sole attribution to concomitant anticancer drugs. Second, mild cutaneous and systemic SDRs (e.g., erythema, peripheral oedema, fatigue) remain robust, indicating these mild toxicities are more likely to be directly associated with pemetrexed itself rather than drug-drug interactions, which provides a more precise reference for clinical management of pemetrexed monotherapy-related adverse reactions. Third, the sensitivity analysis has key limitations: the substantial sample size reduction (from 27,098 to 1,951) markedly diminished statistical power to detect low-frequency SDRs (e.g., immune-mediated endocrine disorders, tubulointerstitial nephritis), potentially leading to false-negative results for rare but clinically meaningful signals. Additionally, the reduced sample size may compromise the stability of signal estimates, as the non-combination subset may not fully represent the broader population of pemetrexed users (e.g., patients with complex comorbidities). Despite these constraints, the consistency of core safety signals (e.g., hematologic toxicity, pyrexia) between the full dataset and the non-combination subset supports the robustness of these key findings. Additionally, only four common combined drugs (including pembrolizumab) were excluded; FAERS coding constraints prevented inclusion of all ICI types (e.g., nivolumab, atezolizumab), precluding complete elimination of ICI-related confounding for immune-mediated SDRs (e.g., colitis, immune-mediated pneumonitis).

Future studies with larger monotherapy cohorts are needed to validate rare signals and enhance the reliability of subgroup-specific safety profiles.

#### 4.3.8 Implications of reporter subgroup analysis

Subgroup analysis by reporter type [healthcare professionals (HCPs) vs. consumers] identified distinct pemetrexed-associated SDR reporting profiles—reflecting spontaneous reporting system (SRS) reporting bias, not true differences in clinical manifestation spectra across populations. HCPs reported severe SDRs (e.g., respiratory failure, cardiac arrest, death) more frequently—due to professional obligations to report severe/fatal outcomes, comprehensive clinical information access, and standardized reporting practices. In contrast, consumer reports focused on non-fatal, subjective SDRs (e.g., fatigue, nausea)—driven by subjective discomfort, with limited awareness/obligation to report severe outcomes (e.g., death), resulting in relative underreporting of severe events. Notably, the inverse association of the f severe events.obligation to re(ROR = 0.80) does not imply lower actual pemetrexed-associated mortality risk in consumer-reported populations. This reflects significant underreporting of death in consumer reports: HCPs are mandated to report all severe events (including death) per clinical standards, while consumer reports rarely include death due to reporter and information source limitations. Thus, the “death” SDR ROR in reporter subgroups only reflects relative reporting frequency differences between groups, not actual clinical risk differences in the study population. This underscores the need to interpret SRS subgroup results in the context of reporting patterns, avoiding overinterpretation as true clinical risk differences.

### 4.4 Time-to-onset

The temporal relationship between pemetrexed administration and SDR-associated clinical manifestations is critical for pharmacovigilance—it identifies risk windows and informs targeted monitoring hypotheses for drug-related toxicities.

Our time-to-onset analysis identified two clinically relevant windows: a primary peak at 0–30 days (broader 0–60-day period) representing the highest-yield phase for intensive monitoring—consistent with early-onset hematologic, gastrointestinal, and acute organ toxicities typical of antifolate chemotherapy A smaller secondary peak at 181–360 days may reflect delayed events linked to cumulative exposure, prolonged treatment, or delayed immune-mediated phenomena—though reporting delays and missing date fields in spontaneous reports may also contribute. Notably, Weibull distribution analysis quantifies these patterns, with parameters offering specific monitoring guidance: (1) Scale parameter (α = 57.99 days): ~63.2% of SDRs occur within 58 days post-treatment, supporting intensified monitoring for the first 8 weeks—this window covers the early peak and most early-onset toxicities, justifying weekly blood work and renal function testing. (2) Shape parameter (β = 0.69 < 1) confirms an ter renal function testingst early-onset toxicities, ju first month—clinical vigilance should be maximized to promptly manage hematologic toxicity, gastrointestinal reactions, and other acute SDRs. After 8 weeks, onset risk gradually decreases (consistent with β < 1), but monitoring frequency should be adjusted for individual tolerance (not discontinued) to cover the 181–360-day delayed peak. For high-risk populations (e.g., elderly patients, those with baseline renal impairment), the intensive monitoring window may be extended beyond 8 weeks per the scale parameter, while upholding the ded beyond 8 l tolerance oxicity, gastrointestinal reactions Practically, these findings support: (1) close early-cycle surveillance (symptoms, blood counts, renal function, infection screening as indicated); (2) continued, targeted long-term follow-up for patients on extended pemetrexed therapy—especially for pulmonary, renal, and endocrine abnormalities.

### 4.5 Limitation

Our study identified significant disproportionate reporting signals (SDRs) for pemetrexed using the FAERS database, but inherent pharmacovigilance limitations restrict causal inference and clinical generalization: FAERS, as a spontaneous reporting system, is prone to underreporting, incomplete documentation, and variable practices that may distort SDR detection; definitive causality cannot be confirmed due to confounders including underlying malignancy, concomitant medications (e.g., ICIs, platinum agents), prior therapies, and unrecorded comorbidities (unlabeled signals like endocrine disorders are hypothesis-generating); pemetrexed monotherapy rarity introduces selection bias when excluding combination regimens, with sensitivity analyses only supporting signal robustness not sole causality; key clinical data gaps (comorbidities, laboratory results, detailed ICI exposure) preclude reliable stratification of immune-mediated events; and overrepresentation of Western/Japanese data limits generalizability to other populations. Notably, FAERS outcome fields reflect reporting practices rather than causal attribution to pemetrexed, so our findings are safety alerts not proven causal harm, warranting validation via prospective multicenter studies, mechanistic research, and well-controlled trials.

## 5 Conclusion

This study systematically analyzed pemetrexed-associated signals of disproportionate reporting signals (SDRs) using FAERS real-world data, confirming established SDRs linked to its known toxicities (hematologic, gastrointestinal, renal) and identifying novel unlabeled signals—particularly immune-mediated endocrine, renal, and dermatologic manifestations. Time-to-onset (TTO) analysis showed most AEs occur within the first 2 months post-treatment, reflecting early toxicity patterns, while subgroup analyses revealed sex- and age-related disparities in SDR reporting that may inform individualized risk mitigation.

Most existing pharmacovigilance research on pemetrexed has focused on known toxicities in small cohorts, with limited real-world data regarding rare or immune-mediated adverse events. Using long-term FAERS data, our study provides a comprehensive safety profile and detects several uncommon immune-related signals not previously clarified. Future research should focus on prospective validation of these signals, mechanistic exploration of immune-mediated organ injury, and personalized monitoring strategies, while accounting for the inherent limitations of spontaneous reporting systems.

## Data Availability

The datasets presented in this study can be found in online repositories. The names of the repository/repositories and accession number(s) can be found in the article/Supplementary material.
