# Supplementary material for: Adverse events profiles of pemetrexed: a Food and Drug Administration Adverse Event Reporting System
Source: Front Med (Lausanne). 2026 Apr 15;13:1750861. doi: 10.3389/fmed.2026.1750861 (PMC13124966; doi:10.3389/fmed.2026.1750861)
Supplement: Supplementary file 1 [file Data_Sheet_1.docx]

The supplementary Table legends

Supplementary Table S1

Two-by-two contingency table for disproportionality analyses.

Supplementary Table S2

Four major algorithms used for signal detection.

Supplementary Table S3

Top 40 most frequent adverse events meeting the positive signal threshold at the PT level from FAERS data

Supplementary Table S4

Top 50 most frequent adverse events for Pemetexed at the preferred term (PT) level in males from FAERS data

Supplementary Table S5

Top 50 most frequent adverse events for Pemetexed at the PT level in females from FAERS data

Supplementary Table S6

Adverse events at the PT level for pemetrexed in patients aged under 18 from FAERS data

Supplementary Table S7

Top 50 most frequent adverse events for pemetrexed at the PT level in patients aged 18 to 65 from FAERS data

Supplementary Table S8

Top 50 most frequent adverse events for pemetrexed at the PT level in patients aged over 65 from FAERS data

Supplementary Table S9

Top 50 most frequent adverse events for pemetrexed excluding common medication co-usage at the PT level from FAERS data

Supplementary Tables

Supplementary Table S1:

Two-by-two contingency table for disproportionality analyses.

|  | Target AEs | Other AEs | Total |
| --- | --- | --- | --- |
| Pemetexed | a | b | a+b |
| Other drugs | c | d | c+d |
| Total | a+c | b+d | a+b+c+d |

Abbreviation: AEs, adverse events; a, number of reports containing both the target drug and target adverse drug reaction; b, number of reports containing other adverse drug reaction of the target drug; c, number of reports containing the target adverse drug reaction of other drugs; d, number of reports containing other drugs and other adverse drug reactions.

Supplementary Table S2:

Four major algorithms used for signal detection.

| Algorithms | Equation | Criteria |
| --- | --- | --- |
| ROR | ROR=ad/b/c | lower limit of 95% CI>1, N≥3 |
|  | 95%CI=e^ln(ROR)±1.96(1/a+1/b+1/c+1/d)^0.5^ |  |
| PRR | PRR=a(c+d)/c/(a+b) | PRR≥2, χ^2^≥4, N≥3 |
|  | χ^2^=[(ad-bc)^2](a+b+c+d)/[(a+b)(c+d)(a+c)(b+d)] |  |
| BCPNN | IC=log_2_a(a+b+c+d)(a+c)(a+b) | IC025>0 |
|  | 95%CI= E(IC) ± 2V(IC)^0.5 |  |
| MGPS | EBGM=a(a+b+c+d)/(a+c)/(a+b) | EBGM05>2 |
|  | 95%CI=e^ln(EBGM)±1.96(1/a+1/b+1/c+1/d)^0.5^ |  |

Abbreviation: a, number of reports containing both the target drug and target adverse drug reaction; b, number of reports containing other adverse drug reaction of the target drug; c, number of reports containing the target adverse drug reaction of other drugs; d, number of reports containing other drugs and other adverse drug reactions. 95%CI, 95% confidence interval; N, the number of reports; χ2, chi-squared; IC, information component; IC025, the lower limit of 95% CI of the IC; E(IC), the IC expectations; V(IC), the variance of IC; EBGM, empirical Bayesian geometric mean; EBGM05, the lower limit of 95% CI of EBGM.

Supplementary Table S3 :

Top 40 most frequent adverse events meeting the positive signal threshold at the PT level from FAERS data

| PT | Case numbers | ROR(95%CI) | PRR(χ^2^) | EBGM(EBGM05) | IC(IC025) |
| --- | --- | --- | --- | --- | --- |
| Malignant neoplasm progression | 2418 | 17.91 ( 17.19 - 18.65 ) | 17.44 ( 36501.7 ) | 16.99 ( 16.42 ) | 4.09 ( 4.03 ) |
| Anaemia | 1733 | 6.33 ( 6.04 - 6.64 ) | 6.22 ( 7548.48 ) | 6.17 ( 5.93 ) | 2.63 ( 2.56 ) |
| Off label use | 1708 | 1.44 ( 1.37 - 1.51 ) | 1.43 ( 224.12 ) | 1.43 ( 1.37 ) | 0.52 ( 0.45 ) |
| Nausea | 1471 | 1.3 ( 1.24 - 1.37 ) | 1.3 ( 100.62 ) | 1.3 ( 1.24 ) | 0.37 ( 0.3 ) |
| Pancytopenia | 1396 | 18.26 ( 17.3 - 19.26 ) | 17.98 ( 21778.16 ) | 17.5 ( 16.74 ) | 4.13 ( 4.05 ) |
| Neutropenia | 1393 | 7.26 ( 6.88 - 7.65 ) | 7.16 ( 7310.2 ) | 7.09 ( 6.78 ) | 2.83 ( 2.75 ) |
| Diarrhoea | 1384 | 1.51 ( 1.44 - 1.6 ) | 1.51 ( 237.06 ) | 1.5 ( 1.44 ) | 0.59 ( 0.51 ) |
| Thrombocytopenia | 1341 | 8.55 ( 8.1 - 9.03 ) | 8.43 ( 8683.22 ) | 8.33 ( 7.96 ) | 3.06 ( 2.98 ) |
| Vomiting | 1135 | 1.7 ( 1.61 - 1.81 ) | 1.69 ( 323.95 ) | 1.69 ( 1.61 ) | 0.76 ( 0.67 ) |
| Febrile neutropenia | 1035 | 11.22 ( 10.55 - 11.94 ) | 11.1 ( 9356.78 ) | 10.92 ( 10.37 ) | 3.45 ( 3.36 ) |
| Disease progression | 1032 | 6.21 ( 5.84 - 6.6 ) | 6.15 ( 4413.46 ) | 6.1 ( 5.79 ) | 2.61 ( 2.52 ) |
| Pyrexia | 1010 | 2.01 ( 1.89 - 2.14 ) | 2 ( 502.63 ) | 1.99 ( 1.89 ) | 0.99 ( 0.9 ) |
| Acute kidney injury | 979 | 4.6 ( 4.32 - 4.9 ) | 4.56 ( 2708.59 ) | 4.53 ( 4.3 ) | 2.18 ( 2.09 ) |
| Dyspnoea | 977 | 1.19 ( 1.12 - 1.27 ) | 1.19 ( 30.19 ) | 1.19 ( 1.13 ) | 0.25 ( 0.16 ) |
| Pneumonia | 966 | 2.11 ( 1.98 - 2.25 ) | 2.1 ( 554.93 ) | 2.09 ( 1.98 ) | 1.07 ( 0.97 ) |
| Asthenia | 880 | 1.62 ( 1.51 - 1.73 ) | 1.61 ( 204.91 ) | 1.61 ( 1.52 ) | 0.69 ( 0.59 ) |
| Rash | 850 | 1.39 ( 1.3 - 1.49 ) | 1.39 ( 92.31 ) | 1.39 ( 1.31 ) | 0.47 ( 0.37 ) |
| General physical health deterioration | 821 | 5.24 ( 4.89 - 5.62 ) | 5.2 ( 2769.35 ) | 5.17 ( 4.88 ) | 2.37 ( 2.27 ) |
| Decreased appetite | 781 | 2.39 ( 2.22 - 2.56 ) | 2.37 ( 621.01 ) | 2.37 ( 2.23 ) | 1.24 ( 1.14 ) |
| Interstitial lung disease | 632 | 9.39 ( 8.68 - 10.16 ) | 9.33 ( 4632.1 ) | 9.2 ( 8.62 ) | 3.2 ( 3.09 ) |
| Platelet count decreased | 590 | 3.83 ( 3.53 - 4.15 ) | 3.81 ( 1217.48 ) | 3.79 ( 3.54 ) | 1.92 ( 1.8 ) |
| Renal failure | 579 | 2.9 ( 2.67 - 3.14 ) | 2.88 ( 710.15 ) | 2.87 ( 2.68 ) | 1.52 ( 1.4 ) |
| Sepsis | 539 | 3.34 ( 3.07 - 3.64 ) | 3.33 ( 874.71 ) | 3.32 ( 3.09 ) | 1.73 ( 1.6 ) |
| Pneumonitis | 539 | 14.69 ( 13.48 - 16 ) | 14.6 ( 6675.21 ) | 14.29 ( 13.3 ) | 3.84 ( 3.71 ) |
| Leukopenia | 535 | 7.5 ( 6.89 - 8.17 ) | 7.46 ( 2959.9 ) | 7.38 ( 6.87 ) | 2.88 ( 2.76 ) |
| Dehydration | 533 | 2.75 ( 2.53 - 3 ) | 2.74 ( 587.97 ) | 2.73 ( 2.54 ) | 1.45 ( 1.32 ) |
| Pulmonary embolism | 495 | 3.53 ( 3.23 - 3.86 ) | 3.51 ( 887.11 ) | 3.5 ( 3.25 ) | 1.81 ( 1.68 ) |
| Pleural effusion | 479 | 5.4 ( 4.94 - 5.91 ) | 5.38 ( 1693.71 ) | 5.34 ( 4.95 ) | 2.42 ( 2.28 ) |
| Mucosal inflammation | 477 | 13.08 ( 11.94 - 14.33 ) | 13.02 ( 5184.69 ) | 12.77 ( 11.83 ) | 3.67 ( 3.54 ) |
| Neoplasm progression | 477 | 9.05 ( 8.26 - 9.9 ) | 9 ( 3346.72 ) | 8.89 ( 8.24 ) | 3.15 ( 3.02 ) |
| White blood cell count decreased | 473 | 3 ( 2.74 - 3.29 ) | 2.99 ( 626.26 ) | 2.98 ( 2.77 ) | 1.58 ( 1.44 ) |
| Myelosuppression | 473 | 14.14 ( 12.91 - 15.49 ) | 14.07 ( 5617.95 ) | 13.78 ( 12.77 ) | 3.78 ( 3.65 ) |
| Constipation | 465 | 1.54 ( 1.4 - 1.69 ) | 1.54 ( 86.97 ) | 1.53 ( 1.42 ) | 0.62 ( 0.48 ) |
| Respiratory failure | 449 | 4.24 ( 3.87 - 4.66 ) | 4.23 ( 1099.4 ) | 4.2 ( 3.89 ) | 2.07 ( 1.93 ) |
| Neutrophil count decreased | 400 | 6.99 ( 6.33 - 7.72 ) | 6.96 ( 2021.51 ) | 6.9 ( 6.35 ) | 2.79 ( 2.64 ) |
| Renal impairment | 394 | 3.32 ( 3 - 3.66 ) | 3.31 ( 631.5 ) | 3.29 ( 3.03 ) | 1.72 ( 1.57 ) |
| Tubulointerstitial nephritis | 380 | 14.47 ( 13.07 - 16.03 ) | 14.41 ( 4637.7 ) | 14.11 ( 12.96 ) | 3.82 ( 3.67 ) |
| Blood creatinine increased | 373 | 3.88 ( 3.5 - 4.29 ) | 3.87 ( 788.38 ) | 3.85 ( 3.53 ) | 1.94 ( 1.79 ) |
| Bone marrow failure | 358 | 11.77 ( 10.6 - 13.07 ) | 11.73 ( 3449.07 ) | 11.53 ( 10.56 ) | 3.53 ( 3.37 ) |
| Haemoglobin decreased | 355 | 2.34 ( 2.11 - 2.6 ) | 2.34 ( 270.87 ) | 2.33 ( 2.14 ) | 1.22 ( 1.07 ) |

Abbreviation: ROR, reporting odds ratio; PRR, proportional reporting ratio; EBGM, empirical Bayesian geometric mean; EBGM05, the lower limit of the 95% CI of EBGM; IC, information component; IC025, the lower limit of the 95% CI of the IC; CI, confidence interval; PT,preferred term.

Supplementary Table S4:

Top 50 most frequent adverse events for Pemetexed at the preferred term (PT) level in males from FAERS data

| PT | Case numbers | ROR(95%CI) | PRR(χ^2^) | EBGM(EBGM05) | IC(IC025) |
| --- | --- | --- | --- | --- | --- |
| Malignant neoplasm progression* | 1,262 | 12.67 ( 11.97 - 13.41 ) | 12.36 ( 12798.94 ) | 12.01 ( 11.45 ) | 3.59 ( 3.5 ) |
| Off label use* | 969 | 1.53 ( 1.43 - 1.63 ) | 1.52 ( 173.24 ) | 1.52 ( 1.44 ) | 0.6 ( 0.51 ) |
| Anaemia* | 956 | 5.73 ( 5.37 - 6.11 ) | 5.64 ( 3606.76 ) | 5.57 ( 5.28 ) | 2.48 ( 2.38 ) |
| Pancytopenia* | 881 | 16.12 ( 15.06 - 17.25 ) | 15.84 ( 11775.45 ) | 15.25 ( 14.41 ) | 3.93 ( 3.83 ) |
| Neutropenia* | 795 | 7.11 ( 6.63 - 7.63 ) | 7.01 ( 4032.85 ) | 6.9 ( 6.51 ) | 2.79 ( 2.68 ) |
| Thrombocytopenia* | 782 | 7.25 ( 6.75 - 7.79 ) | 7.15 ( 4069.91 ) | 7.04 ( 6.63 ) | 2.81 ( 2.71 ) |
| Diarrhoea* | 763 | 1.57 ( 1.46 - 1.69 ) | 1.56 ( 156.33 ) | 1.56 ( 1.47 ) | 0.64 ( 0.54 ) |
| Nausea* | 651 | 1.42 ( 1.31 - 1.53 ) | 1.41 ( 79.07 ) | 1.41 ( 1.32 ) | 0.5 ( 0.38 ) |
| Pyrexia* | 627 | 1.98 ( 1.83 - 2.14 ) | 1.97 ( 298.04 ) | 1.96 ( 1.84 ) | 0.97 ( 0.86 ) |
| Pneumonia* | 625 | 2.12 ( 1.96 - 2.29 ) | 2.1 ( 362.29 ) | 2.1 ( 1.96 ) | 1.07 ( 0.95 ) |
| Dyspnoea* | 620 | 1.38 ( 1.27 - 1.49 ) | 1.37 ( 63.1 ) | 1.37 ( 1.28 ) | 0.46 ( 0.34 ) |
| Febrile neutropenia* | 593 | 9.09 ( 8.38 - 9.87 ) | 8.99 ( 4122.94 ) | 8.81 ( 8.23 ) | 3.14 ( 3.02 ) |
| General physical health deterioration* | 570 | 5.72 ( 5.27 - 6.22 ) | 5.67 ( 2163.25 ) | 5.6 ( 5.22 ) | 2.49 ( 2.36 ) |
| Death | 555 | 0.61 ( 0.57 - 0.67 ) | 0.62 ( 132.03 ) | 0.62 ( 0.58 ) | -0.69 ( -0.81 ) |
| Acute kidney injury* | 552 | 3.58 ( 3.29 - 3.9 ) | 3.55 ( 1005.87 ) | 3.53 ( 3.29 ) | 1.82 ( 1.69 ) |
| Vomiting* | 515 | 1.7 ( 1.55 - 1.85 ) | 1.69 ( 144.81 ) | 1.69 ( 1.57 ) | 0.75 ( 0.63 ) |
| Disease progression* | 501 | 4.63 ( 4.23 - 5.05 ) | 4.59 ( 1391.96 ) | 4.54 ( 4.22 ) | 2.18 ( 2.05 ) |
| Fatigue | 498 | 0.87 ( 0.8 - 0.96 ) | 0.88 ( 8.81 ) | 0.88 ( 0.81 ) | -0.19 ( -0.32 ) |
| Asthenia* | 486 | 1.53 ( 1.4 - 1.67 ) | 1.52 ( 87.42 ) | 1.52 ( 1.41 ) | 0.6 ( 0.47 ) |
| Rash* | 468 | 1.49 ( 1.36 - 1.63 ) | 1.48 ( 73.3 ) | 1.48 ( 1.37 ) | 0.56 ( 0.43 ) |
| Interstitial lung disease* | 456 | 8.96 ( 8.16 - 9.83 ) | 8.88 ( 3120.07 ) | 8.7 ( 8.05 ) | 3.12 ( 2.98 ) |
| Decreased appetite* | 456 | 2.28 ( 2.08 - 2.5 ) | 2.27 ( 322.72 ) | 2.26 ( 2.09 ) | 1.18 ( 1.04 ) |
| Drug ineffective | 404 | 0.41 ( 0.38 - 0.46 ) | 0.42 ( 332.09 ) | 0.42 ( 0.39 ) | -1.25 ( -1.4 ) |
| Platelet count decreased* | 368 | 3.51 ( 3.17 - 3.9 ) | 3.49 ( 650.88 ) | 3.47 ( 3.19 ) | 1.8 ( 1.64 ) |
| Sepsis* | 339 | 2.96 ( 2.66 - 3.3 ) | 2.95 ( 434.72 ) | 2.94 ( 2.68 ) | 1.55 ( 1.4 ) |
| Renal failure* | 326 | 2.15 ( 1.93 - 2.4 ) | 2.15 ( 199.03 ) | 2.14 ( 1.95 ) | 1.1 ( 0.94 ) |
| Dehydration* | 300 | 2.52 ( 2.25 - 2.83 ) | 2.51 ( 272.54 ) | 2.5 ( 2.28 ) | 1.32 ( 1.16 ) |
| Leukopenia* | 298 | 6.94 ( 6.18 - 7.78 ) | 6.9 ( 1478.13 ) | 6.8 ( 6.17 ) | 2.76 ( 2.6 ) |
| Mucosal inflammation* | 292 | 13.38 ( 11.9 - 15.04 ) | 13.3 ( 3212.3 ) | 12.89 ( 11.69 ) | 3.69 ( 3.52 ) |
| Pleural effusion* | 285 | 4.58 ( 4.07 - 5.15 ) | 4.56 ( 783.68 ) | 4.52 ( 4.1 ) | 2.18 ( 2 ) |
| Respiratory failure* | 284 | 3.52 ( 3.13 - 3.96 ) | 3.51 ( 505.4 ) | 3.48 ( 3.16 ) | 1.8 ( 1.63 ) |
| Pneumonitis* | 276 | 11.58 ( 10.27 - 13.05 ) | 11.52 ( 2574.64 ) | 11.21 ( 10.14 ) | 3.49 ( 3.31 ) |
| White blood cell count decreased* | 272 | 3.16 ( 2.8 - 3.56 ) | 3.15 ( 396.32 ) | 3.13 ( 2.83 ) | 1.65 ( 1.47 ) |
| Pulmonary embolism* | 250 | 3.17 ( 2.79 - 3.59 ) | 3.15 ( 365.43 ) | 3.14 ( 2.83 ) | 1.65 ( 1.47 ) |
| Septic shock* | 245 | 5.28 ( 4.66 - 6 ) | 5.26 ( 835.17 ) | 5.2 ( 4.68 ) | 2.38 ( 2.19 ) |
| Hypotension* | 244 | 1.24 ( 1.1 - 1.41 ) | 1.24 ( 11.66 ) | 1.24 ( 1.12 ) | 0.31 ( 0.13 ) |
| Constipation* | 235 | 1.46 ( 1.28 - 1.66 ) | 1.45 ( 33.35 ) | 1.45 ( 1.3 ) | 0.54 ( 0.35 ) |
| Neoplasm progression* | 232 | 8.78 ( 7.71 - 10.01 ) | 8.75 ( 1557.08 ) | 8.57 ( 7.69 ) | 3.1 ( 2.91 ) |
| Renal impairment* | 231 | 2.63 ( 2.31 - 2.99 ) | 2.62 ( 229.78 ) | 2.61 ( 2.34 ) | 1.38 ( 1.19 ) |
| Neutrophil count decreased* | 229 | 6.21 ( 5.45 - 7.08 ) | 6.18 ( 980.2 ) | 6.1 ( 5.47 ) | 2.61 ( 2.42 ) |
| Haemoglobin decreased* | 211 | 2.08 ( 1.81 - 2.38 ) | 2.07 ( 116.44 ) | 2.06 ( 1.84 ) | 1.05 ( 0.85 ) |
| Oedema peripheral* | 207 | 2.03 ( 1.77 - 2.33 ) | 2.03 ( 107.32 ) | 2.02 ( 1.8 ) | 1.02 ( 0.81 ) |
| Bone marrow failure* | 205 | 10.42 ( 9.06 - 11.97 ) | 10.38 ( 1691.87 ) | 10.13 ( 9.02 ) | 3.34 ( 3.14 ) |
| Weight decreased | 199 | 0.8 ( 0.69 - 0.91 ) | 0.8 ( 10.41 ) | 0.8 ( 0.71 ) | -0.33 ( -0.53 ) |
| Colitis* | 191 | 6.32 ( 5.48 - 7.29 ) | 6.3 ( 838.07 ) | 6.21 ( 5.51 ) | 2.64 ( 2.42 ) |
| Myelosuppression* | 189 | 10.36 ( 8.97 - 11.98 ) | 10.32 ( 1550.62 ) | 10.08 ( 8.93 ) | 3.33 ( 3.12 ) |
| Blood creatinine increased* | 185 | 2.39 ( 2.07 - 2.76 ) | 2.38 ( 148.09 ) | 2.38 ( 2.11 ) | 1.25 ( 1.04 ) |
| Atrial fibrillation* | 185 | 1.75 ( 1.51 - 2.02 ) | 1.75 ( 58.81 ) | 1.74 ( 1.54 ) | 0.8 ( 0.59 ) |
| Malaise | 180 | 0.6 ( 0.52 - 0.7 ) | 0.6 ( 46.74 ) | 0.61 ( 0.54 ) | -0.72 ( -0.94 ) |
| Condition aggravated | 169 | 0.77 ( 0.66 - 0.89 ) | 0.77 ( 11.66 ) | 0.77 ( 0.68 ) | -0.38 ( -0.6 ) |

Abbreviation: Asterisks (*) indicate statistically significant signals in algorithm; ROR, reporting odds ratio; PRR, proportional reporting ratio; EBGM, empirical Bayesian geometric mean; EBGM05, the lower limit of the 95% CI of EBGM; IC, information component; IC025, the lower limit of the 95% CI of the IC; CI, confidence interval; PT,preferred term; AEs, adverse events.

Supplementary Table S5:

Top 50 most frequent adverse events for Pemetexed at the PT level in females from FAERS data

| PT | Case numbers | ROR(95%CI) | PRR(χ^2^) | EBGM(EBGM05) | IC(IC025) |
| --- | --- | --- | --- | --- | --- |
| Malignant neoplasm progression* | 925 | 24.12 ( 22.58 - 25.78 ) | 23.45 ( 19432.97 ) | 22.92 ( 21.68 ) | 4.52 ( 4.42 ) |
| Nausea* | 675 | 1.39 ( 1.28 - 1.5 ) | 1.38 ( 71.03 ) | 1.38 ( 1.29 ) | 0.46 ( 0.35 ) |
| Off label use* | 674 | 1.75 ( 1.62 - 1.88 ) | 1.73 ( 209.49 ) | 1.73 ( 1.62 ) | 0.79 ( 0.68 ) |
| Anaemia* | 579 | 6.37 ( 5.86 - 6.91 ) | 6.27 ( 2554.55 ) | 6.23 ( 5.82 ) | 2.64 ( 2.52 ) |
| Vomiting* | 505 | 1.85 ( 1.69 - 2.02 ) | 1.83 ( 192.68 ) | 1.83 ( 1.7 ) | 0.87 ( 0.74 ) |
| Diarrhoea* | 502 | 1.44 ( 1.32 - 1.57 ) | 1.43 ( 66.03 ) | 1.43 ( 1.33 ) | 0.52 ( 0.39 ) |
| Pancytopenia* | 460 | 19.96 ( 18.19 - 21.9 ) | 19.68 ( 8000.2 ) | 19.31 ( 17.86 ) | 4.27 ( 4.13 ) |
| Fatigue | 421 | 0.97 ( 0.88 - 1.06 ) | 0.97 ( 0.51 ) | 0.97 ( 0.89 ) | -0.05 ( -0.19 ) |
| Disease progression* | 417 | 9.04 ( 8.21 - 9.97 ) | 8.94 ( 2917.03 ) | 8.86 ( 8.17 ) | 3.15 ( 3.01 ) |
| Neutropenia* | 406 | 7.26 ( 6.58 - 8.01 ) | 7.18 ( 2148.31 ) | 7.14 ( 6.57 ) | 2.84 ( 2.69 ) |
| Thrombocytopenia* | 391 | 9.32 ( 8.43 - 10.3 ) | 9.21 ( 2840.09 ) | 9.14 ( 8.4 ) | 3.19 ( 3.04 ) |
| Acute kidney injury* | 352 | 6.67 ( 6.01 - 7.41 ) | 6.61 ( 1667.27 ) | 6.57 ( 6.02 ) | 2.72 ( 2.56 ) |
| Pyrexia* | 334 | 1.93 ( 1.73 - 2.15 ) | 1.92 ( 148.2 ) | 1.92 ( 1.75 ) | 0.94 ( 0.78 ) |
| Asthenia* | 331 | 1.65 ( 1.48 - 1.84 ) | 1.64 ( 83.45 ) | 1.64 ( 1.5 ) | 0.71 ( 0.56 ) |
| Febrile neutropenia* | 312 | 12.9 ( 11.53 - 14.43 ) | 12.78 ( 3346.46 ) | 12.63 ( 11.5 ) | 3.66 ( 3.49 ) |
| Drug ineffective | 309 | 0.46 ( 0.41 - 0.51 ) | 0.46 ( 199.17 ) | 0.46 ( 0.42 ) | -1.12 ( -1.28 ) |
| Death | 309 | 0.98 ( 0.88 - 1.1 ) | 0.98 ( 0.09 ) | 0.98 ( 0.9 ) | -0.02 ( -0.19 ) |
| Dyspnoea | 303 | 0.98 ( 0.87 - 1.1 ) | 0.98 ( 0.13 ) | 0.98 ( 0.89 ) | -0.03 ( -0.2 ) |
| Rash* | 282 | 1.22 ( 1.08 - 1.37 ) | 1.22 ( 10.98 ) | 1.22 ( 1.1 ) | 0.28 ( 0.11 ) |
| Pneumonia* | 269 | 1.75 ( 1.55 - 1.97 ) | 1.74 ( 84.76 ) | 1.74 ( 1.57 ) | 0.8 ( 0.62 ) |
| General physical health deterioration* | 237 | 4.57 ( 4.02 - 5.19 ) | 4.54 ( 652.08 ) | 4.52 ( 4.06 ) | 2.18 ( 1.99 ) |
| Decreased appetite* | 228 | 1.98 ( 1.74 - 2.25 ) | 1.97 ( 109.03 ) | 1.97 ( 1.76 ) | 0.98 ( 0.79 ) |
| Renal failure* | 216 | 4.05 ( 3.54 - 4.63 ) | 4.03 ( 490.72 ) | 4.02 ( 3.59 ) | 2.01 ( 1.81 ) |
| Dehydration* | 209 | 3 ( 2.62 - 3.44 ) | 2.99 ( 276.82 ) | 2.99 ( 2.66 ) | 1.58 ( 1.38 ) |
| Pulmonary embolism* | 208 | 4.04 ( 3.52 - 4.63 ) | 4.02 ( 470.1 ) | 4 ( 3.57 ) | 2 ( 1.8 ) |
| Neoplasm progression* | 207 | 9.93 ( 8.66 - 11.4 ) | 9.87 ( 1635.26 ) | 9.78 ( 8.72 ) | 3.29 ( 3.09 ) |
| Tubulointerstitial nephritis* | 201 | 30.94 ( 26.87 - 35.62 ) | 30.75 ( 5606.76 ) | 29.83 ( 26.51 ) | 4.9 ( 4.69 ) |
| Pneumonitis* | 183 | 16.93 ( 14.62 - 19.61 ) | 16.84 ( 2680.68 ) | 16.57 ( 14.66 ) | 4.05 ( 3.84 ) |
| Constipation* | 179 | 1.62 ( 1.4 - 1.87 ) | 1.61 ( 41.95 ) | 1.61 ( 1.43 ) | 0.69 ( 0.47 ) |
| Platelet count decreased* | 173 | 3.68 ( 3.17 - 4.27 ) | 3.66 ( 334.1 ) | 3.65 ( 3.22 ) | 1.87 ( 1.65 ) |
| Pleural effusion* | 161 | 5.67 ( 4.86 - 6.63 ) | 5.65 ( 613.21 ) | 5.62 ( 4.94 ) | 2.49 ( 2.26 ) |
| Mucosal inflammation* | 154 | 13.93 ( 11.88 - 16.34 ) | 13.87 ( 1813.05 ) | 13.68 ( 11.97 ) | 3.77 ( 3.54 ) |
| Hypokalaemia* | 153 | 6.35 ( 5.41 - 7.44 ) | 6.32 ( 681.21 ) | 6.29 ( 5.5 ) | 2.65 ( 2.42 ) |
| White blood cell count decreased* | 153 | 2.5 ( 2.14 - 2.94 ) | 2.5 ( 137.21 ) | 2.49 ( 2.18 ) | 1.32 ( 1.08 ) |
| Headache | 151 | 0.38 ( 0.32 - 0.44 ) | 0.38 ( 153.61 ) | 0.38 ( 0.33 ) | -1.39 ( -1.63 ) |
| Blood creatinine increased* | 150 | 5.95 ( 5.06 - 6.98 ) | 5.92 ( 610.43 ) | 5.89 ( 5.15 ) | 2.56 ( 2.32 ) |
| Sepsis* | 149 | 3.2 ( 2.72 - 3.76 ) | 3.19 ( 223.46 ) | 3.18 ( 2.78 ) | 1.67 ( 1.43 ) |
| Leukopenia* | 131 | 5.71 ( 4.8 - 6.78 ) | 5.69 ( 503.47 ) | 5.66 ( 4.9 ) | 2.5 ( 2.25 ) |
| Abdominal pain | 130 | 0.97 ( 0.82 - 1.16 ) | 0.97 ( 0.1 ) | 0.97 ( 0.84 ) | -0.04 ( -0.29 ) |
| Bone marrow failure* | 130 | 14.39 ( 12.1 - 17.11 ) | 14.33 ( 1589.11 ) | 14.14 ( 12.23 ) | 3.82 ( 3.57 ) |
| Malaise | 129 | 0.48 ( 0.4 - 0.57 ) | 0.48 ( 74.1 ) | 0.48 ( 0.41 ) | -1.06 ( -1.32 ) |
| Pruritus | 128 | 0.62 ( 0.52 - 0.74 ) | 0.62 ( 29.83 ) | 0.62 ( 0.54 ) | -0.69 ( -0.94 ) |
| Myelosuppression* | 128 | 11.77 ( 9.88 - 14.01 ) | 11.72 ( 1240.92 ) | 11.59 ( 10.02 ) | 3.54 ( 3.28 ) |
| Cough | 127 | 0.81 ( 0.68 - 0.96 ) | 0.81 ( 5.85 ) | 0.81 ( 0.7 ) | -0.31 ( -0.56 ) |
| Renal impairment* | 126 | 3.94 ( 3.3 - 4.69 ) | 3.93 ( 273.95 ) | 3.91 ( 3.38 ) | 1.97 ( 1.71 ) |
| Haemoglobin decreased* | 126 | 2.59 ( 2.18 - 3.09 ) | 2.59 ( 122.51 ) | 2.58 ( 2.23 ) | 1.37 ( 1.11 ) |
| Therapy partial responder* | 126 | 29.5 ( 24.7 - 35.23 ) | 29.38 ( 3352.73 ) | 28.54 ( 24.6 ) | 4.84 ( 4.57 ) |
| Respiratory failure* | 117 | 3.82 ( 3.18 - 4.58 ) | 3.81 ( 241.52 ) | 3.8 ( 3.26 ) | 1.92 ( 1.66 ) |
| Neutrophil count decreased* | 117 | 6.22 ( 5.19 - 7.47 ) | 6.2 ( 507.71 ) | 6.17 ( 5.3 ) | 2.63 ( 2.36 ) |
| Dizziness | 115 | 0.39 ( 0.33 - 0.47 ) | 0.4 ( 106.18 ) | 0.4 ( 0.34 ) | -1.33 ( -1.6 ) |

Abbreviation: Asterisks (*) indicate statistically significant signals in algorithm; ROR, reporting odds ratio; PRR, proportional reporting ratio; EBGM, empirical Bayesian geometric mean; EBGM05, the lower limit of the 95% CI of EBGM; IC, information component; IC025, the lower limit of the 95% CI of the IC; CI, confidence interval; PT,preferred term; AEs, adverse events.

Supplementary Table S6:

Adverse events at the PT level for pemetrexed in patients aged under 18 from FAERS data

| PT | Case numbers | ROR(95%CI) | PRR(χ^2^) | EBGM(EBGM05) | IC(IC025) |
| --- | --- | --- | --- | --- | --- |
| Disease progression* | 10 | 30.43 ( 16.13 - 57.39 ) | 29.17 ( 271.5 ) | 29.07 ( 17.1 ) | 4.86 ( 3.97 ) |
| Drug ineffective | 8 | 1.71 ( 0.84 - 3.45 ) | 1.68 ( 2.26 ) | 1.68 ( 0.93 ) | 0.75 ( -0.23 ) |
| Nausea* | 8 | 4.74 ( 2.34 - 9.6 ) | 4.61 ( 22.79 ) | 4.61 ( 2.56 ) | 2.2 ( 1.22 ) |
| Febrile neutropenia* | 6 | 7.73 ( 3.43 - 17.39 ) | 7.55 ( 34.2 ) | 7.55 ( 3.83 ) | 2.92 ( 1.81 ) |
| Renal impairment* | 6 | 25.74 ( 11.43 - 57.97 ) | 25.11 ( 138.62 ) | 25.04 ( 12.69 ) | 4.65 ( 3.54 ) |
| Adrenal insufficiency* | 6 | 63.52 ( 28.16 - 143.3 ) | 61.92 ( 357.1 ) | 61.47 ( 31.12 ) | 5.94 ( 4.83 ) |
| Peripheral sensory neuropathy* | 5 | 410.12 ( 165.63 - 1015.46 ) | 401.37 ( 1905.39 ) | 383.01 ( 179.36 ) | 8.58 ( 7.36 ) |
| Hepatic function abnormal* | 5 | 25.05 ( 10.32 - 60.85 ) | 24.54 ( 112.67 ) | 24.47 ( 11.65 ) | 4.61 ( 3.42 ) |
| Pyrexia | 5 | 1.72 ( 0.71 - 4.18 ) | 1.71 ( 1.48 ) | 1.71 ( 0.81 ) | 0.77 ( -0.42 ) |
| Malignant neoplasm progression* | 5 | 29.35 ( 12.08 - 71.31 ) | 28.75 ( 133.56 ) | 28.65 ( 13.63 ) | 4.84 ( 3.65 ) |
| Decreased appetite* | 4 | 5.05 ( 1.88 - 13.57 ) | 4.98 ( 12.75 ) | 4.98 ( 2.18 ) | 2.31 ( 1.01 ) |
| Myelosuppression* | 4 | 22.72 ( 8.45 - 61.14 ) | 22.35 ( 81.43 ) | 22.3 ( 9.74 ) | 4.48 ( 3.17 ) |
| Immune-mediated hepatic disorder* | 4 | 353.89 ( 129.08 - 970.21 ) | 347.86 ( 1328.17 ) | 333.98 ( 143.63 ) | 8.38 ( 7.05 ) |
| Non-small cell lung cancer* | 4 | 1171.54 ( 408.59 - 3359.12 ) | 1151.53 ( 4040.84 ) | 1012.07 ( 419.21 ) | 9.98 ( 8.59 ) |
| Neuralgia* | 4 | 125.35 ( 46.32 - 339.26 ) | 123.23 ( 477.95 ) | 121.45 ( 52.79 ) | 6.92 ( 5.61 ) |
| Fatigue* | 4 | 3.2 ( 1.19 - 8.6 ) | 3.16 ( 5.94 ) | 3.16 ( 1.38 ) | 1.66 ( 0.36 ) |
| Body temperature decreased* | 4 | 83.46 ( 30.91 - 225.34 ) | 82.05 ( 317.2 ) | 81.26 ( 35.4 ) | 6.34 ( 5.03 ) |
| Cholestasis* | 3 | 32.98 ( 10.54 - 103.23 ) | 32.57 ( 91.48 ) | 32.45 ( 12.49 ) | 5.02 ( 3.56 ) |
| Loss of personal independence in daily activities* | 3 | 45.78 ( 14.61 - 143.44 ) | 45.21 ( 129.03 ) | 44.97 ( 17.3 ) | 5.49 ( 4.03 ) |
| Pneumonia* | 3 | 3.05 ( 0.98 - 9.52 ) | 3.02 ( 4.07 ) | 3.02 ( 1.16 ) | 1.59 ( 0.14 ) |
| Neutropenia* | 3 | 4.58 ( 1.47 - 14.31 ) | 4.54 ( 8.29 ) | 4.53 ( 1.75 ) | 2.18 ( 0.73 ) |
| Oedema* | 3 | 19.38 ( 6.2 - 60.62 ) | 19.15 ( 51.52 ) | 19.11 ( 7.36 ) | 4.26 ( 2.8 ) |
| Therapy partial responder* | 3 | 39.57 ( 12.63 - 123.91 ) | 39.07 ( 110.81 ) | 38.9 ( 14.96 ) | 5.28 ( 3.82 ) |
| Off label use | 2 | 0.32 ( 0.08 - 1.27 ) | 0.32 ( 2.94 ) | 0.32 ( 0.1 ) | -1.64 ( -3.31 ) |
| Rash | 2 | 0.95 ( 0.24 - 3.83 ) | 0.95 ( 0 ) | 0.95 ( 0.3 ) | -0.07 ( -1.75 ) |
| Malaise | 2 | 2.19 ( 0.54 - 8.8 ) | 2.18 ( 1.28 ) | 2.18 ( 0.68 ) | 1.12 ( -0.55 ) |
| Therapeutic product effect incomplete* | 2 | 12.96 ( 3.22 - 52.17 ) | 12.85 ( 21.84 ) | 12.84 ( 4 ) | 3.68 ( 2 ) |
| Diarrhoea | 2 | 1.21 ( 0.3 - 4.88 ) | 1.21 ( 0.07 ) | 1.21 ( 0.38 ) | 0.28 ( -1.4 ) |
| Arthralgia | 2 | 2.93 ( 0.73 - 11.79 ) | 2.91 ( 2.52 ) | 2.91 ( 0.91 ) | 1.54 ( -0.13 ) |
| Proteinuria* | 2 | 16.22 ( 4.03 - 65.31 ) | 16.09 ( 28.26 ) | 16.06 ( 5 ) | 4.01 ( 2.33 ) |
| General physical health deterioration* | 2 | 6.71 ( 1.67 - 27.01 ) | 6.66 ( 9.63 ) | 6.66 ( 2.08 ) | 2.74 ( 1.06 ) |
| Skin disorder* | 2 | 20.5 ( 5.09 - 82.62 ) | 20.34 ( 36.7 ) | 20.29 ( 6.32 ) | 4.34 ( 2.66 ) |
| Leukopenia* | 2 | 9.03 ( 2.24 - 36.33 ) | 8.96 ( 14.14 ) | 8.95 ( 2.79 ) | 3.16 ( 1.48 ) |
| Pulmonary toxicity* | 2 | 114.56 ( 28.21 - 465.13 ) | 113.59 ( 220.21 ) | 112.07 ( 34.7 ) | 6.81 ( 5.12 ) |
| Liver disorder* | 2 | 16.31 ( 4.05 - 65.69 ) | 16.18 ( 28.44 ) | 16.15 ( 5.03 ) | 4.01 ( 2.33 ) |
| Cortisol decreased* | 2 | 187.11 ( 45.81 - 764.23 ) | 185.52 ( 359.1 ) | 181.51 ( 55.92 ) | 7.5 ( 5.8 ) |
| Disease recurrence* | 2 | 10.39 ( 2.58 - 41.82 ) | 10.31 ( 16.8 ) | 10.3 ( 3.21 ) | 3.36 ( 1.69 ) |
| Pneumonia bacterial* | 2 | 55.39 ( 13.71 - 223.81 ) | 54.92 ( 105.21 ) | 54.57 ( 16.96 ) | 5.77 ( 4.09 ) |
| Alveolar rhabdomyosarcoma* | 2 | 990.64 ( 227.59 - 4312.12 ) | 982.19 ( 1754.04 ) | 878.9 ( 256.72 ) | 9.78 ( 7.98 ) |
| Cholelithiasis | 1 | 10.02 ( 1.4 - 71.55 ) | 9.99 ( 8.08 ) | 9.98 ( 1.93 ) | 3.32 ( 1.27 ) |
| Hepatocellular injury | 1 | 10.31 ( 1.44 - 73.58 ) | 10.27 ( 8.36 ) | 10.26 ( 1.98 ) | 3.36 ( 1.31 ) |
| Prothrombin level decreased | 1 | 119.77 ( 16.57 - 865.86 ) | 119.27 ( 115.63 ) | 117.6 ( 22.47 ) | 6.88 ( 4.8 ) |
| Clostridium difficile colitis | 1 | 13.45 ( 1.88 - 96.07 ) | 13.4 ( 11.46 ) | 13.38 ( 2.58 ) | 3.74 ( 1.69 ) |
| Malnutrition | 1 | 15.21 ( 2.13 - 108.65 ) | 15.15 ( 13.2 ) | 15.13 ( 2.92 ) | 3.92 ( 1.87 ) |
| Staphylococcal bacteraemia | 1 | 27.49 ( 3.84 - 196.58 ) | 27.37 ( 25.33 ) | 27.29 ( 5.26 ) | 4.77 ( 2.71 ) |
| Shock | 1 | 6.36 ( 0.89 - 45.36 ) | 6.33 ( 4.49 ) | 6.33 ( 1.22 ) | 2.66 ( 0.61 ) |
| Urinary tract infection | 1 | 4.44 ( 0.62 - 31.66 ) | 4.42 ( 2.65 ) | 4.42 ( 0.85 ) | 2.14 ( 0.09 ) |
| Osteomyelitis | 1 | 26.28 ( 3.67 - 187.92 ) | 26.17 ( 24.14 ) | 26.09 ( 5.03 ) | 4.71 ( 2.65 ) |
| Hypothyroidism | 1 | 10.79 ( 1.51 - 77 ) | 10.74 ( 8.83 ) | 10.73 ( 2.07 ) | 3.42 ( 1.37 ) |
| Metastases to meninges | 1 | 199.62 ( 27.36 - 1456.49 ) | 198.78 ( 192.21 ) | 194.18 ( 36.81 ) | 7.6 ( 5.51 ) |

Abbreviation: Asterisks (*) indicate statistically significant signals in algorithm; ROR, reporting odds ratio; PRR, proportional reporting ratio; EBGM, empirical Bayesian geometric mean; EBGM05, the lower limit of the 95% CI of EBGM; IC, information component; IC025, the lower limit of the 95% CI of the IC; CI, confidence interval; PT, preferred term.

Supplementary Table S7:

Top 50 most frequent adverse events for pemetrexed at the PT level in patients aged 18 to 65 from FAERS data

| PT | Case numbers | ROR(95%CI) | PRR(χ^2^) | EBGM(EBGM05) | IC(IC025) |
| --- | --- | --- | --- | --- | --- |
| Malignant neoplasm progression* | 934 | 22.71 ( 21.25 - 24.26 ) | 22.1 ( 18192.91 ) | 21.37 ( 20.22 ) | 4.42 ( 4.32 ) |
| Off label use* | 667 | 1.74 ( 1.61 - 1.88 ) | 1.72 ( 204.43 ) | 1.72 ( 1.61 ) | 0.78 ( 0.67 ) |
| Anaemia* | 612 | 6.77 ( 6.25 - 7.34 ) | 6.67 ( 2924.96 ) | 6.61 ( 6.18 ) | 2.72 ( 2.61 ) |
| Nausea* | 610 | 1.23 ( 1.13 - 1.33 ) | 1.23 ( 25.59 ) | 1.22 ( 1.15 ) | 0.29 ( 0.17 ) |
| Vomiting* | 539 | 1.9 ( 1.74 - 2.06 ) | 1.88 ( 223.82 ) | 1.88 ( 1.75 ) | 0.91 ( 0.78 ) |
| Disease progression* | 510 | 9.24 ( 8.46 - 10.09 ) | 9.11 ( 3637.52 ) | 9 ( 8.36 ) | 3.17 ( 3.04 ) |
| Diarrhoea* | 503 | 1.54 ( 1.41 - 1.68 ) | 1.53 ( 92.65 ) | 1.53 ( 1.42 ) | 0.61 ( 0.48 ) |
| Pancytopenia* | 492 | 17.3 ( 15.81 - 18.93 ) | 17.06 ( 7245.32 ) | 16.63 ( 15.42 ) | 4.06 ( 3.92 ) |
| Neutropenia* | 450 | 5.7 ( 5.2 - 6.26 ) | 5.64 ( 1706.57 ) | 5.6 ( 5.18 ) | 2.49 ( 2.35 ) |
| Thrombocytopenia* | 441 | 7.87 ( 7.16 - 8.65 ) | 7.77 ( 2575.93 ) | 7.69 ( 7.11 ) | 2.94 ( 2.8 ) |
| Acute kidney injury* | 430 | 6.18 ( 5.62 - 6.8 ) | 6.12 ( 1826.48 ) | 6.07 ( 5.6 ) | 2.6 ( 2.46 ) |
| Drug ineffective | 377 | 0.66 ( 0.59 - 0.73 ) | 0.66 ( 66.88 ) | 0.66 ( 0.61 ) | -0.6 ( -0.75 ) |
| Dyspnoea* | 374 | 1.22 ( 1.1 - 1.35 ) | 1.22 ( 14.99 ) | 1.22 ( 1.12 ) | 0.29 ( 0.14 ) |
| Pyrexia* | 359 | 1.61 ( 1.45 - 1.79 ) | 1.61 ( 82.88 ) | 1.61 ( 1.47 ) | 0.68 ( 0.53 ) |
| Fatigue | 332 | 0.74 ( 0.66 - 0.82 ) | 0.74 ( 30.08 ) | 0.74 ( 0.68 ) | -0.43 ( -0.59 ) |
| Pneumonia* | 330 | 2.26 ( 2.03 - 2.52 ) | 2.25 ( 229.45 ) | 2.25 ( 2.05 ) | 1.17 ( 1.01 ) |
| General physical health deterioration* | 326 | 6.58 ( 5.89 - 7.34 ) | 6.52 ( 1510.15 ) | 6.46 ( 5.9 ) | 2.69 ( 2.53 ) |
| Asthenia* | 297 | 1.52 ( 1.36 - 1.71 ) | 1.52 ( 52.45 ) | 1.52 ( 1.38 ) | 0.6 ( 0.43 ) |
| Death* | 294 | 1.26 ( 1.12 - 1.41 ) | 1.25 ( 15.12 ) | 1.25 ( 1.14 ) | 0.33 ( 0.16 ) |
| Febrile neutropenia* | 294 | 8.99 ( 8 - 10.09 ) | 8.91 ( 2038.77 ) | 8.8 ( 7.99 ) | 3.14 ( 2.97 ) |
| Rash* | 281 | 1.25 ( 1.11 - 1.4 ) | 1.24 ( 13.45 ) | 1.24 ( 1.13 ) | 0.31 ( 0.14 ) |
| Decreased appetite* | 239 | 2.18 ( 1.92 - 2.47 ) | 2.17 ( 150.69 ) | 2.17 ( 1.95 ) | 1.11 ( 0.93 ) |
| Pulmonary embolism* | 226 | 3.63 ( 3.18 - 4.14 ) | 3.61 ( 425.14 ) | 3.6 ( 3.22 ) | 1.85 ( 1.65 ) |
| Tubulointerstitial nephritis* | 207 | 22.1 ( 19.23 - 25.4 ) | 21.97 ( 4003.76 ) | 21.26 ( 18.92 ) | 4.41 ( 4.21 ) |
| Pleural effusion* | 201 | 6.59 ( 5.73 - 7.57 ) | 6.55 ( 937.26 ) | 6.5 ( 5.78 ) | 2.7 ( 2.5 ) |
| Renal failure* | 197 | 3.08 ( 2.68 - 3.55 ) | 3.07 ( 274.2 ) | 3.06 ( 2.72 ) | 1.61 ( 1.41 ) |
| Neoplasm progression* | 191 | 9.44 ( 8.18 - 10.9 ) | 9.39 ( 1411.88 ) | 9.27 ( 8.22 ) | 3.21 ( 3 ) |
| Sepsis* | 187 | 3.35 ( 2.9 - 3.87 ) | 3.33 ( 304.39 ) | 3.32 ( 2.94 ) | 1.73 ( 1.52 ) |
| Mucosal inflammation* | 186 | 14.08 ( 12.17 - 16.29 ) | 14.01 ( 2198.4 ) | 13.72 ( 12.15 ) | 3.78 ( 3.56 ) |
| Pneumonitis* | 185 | 15.48 ( 13.38 - 17.92 ) | 15.4 ( 2432.49 ) | 15.06 ( 13.32 ) | 3.91 ( 3.7 ) |
| Constipation* | 174 | 1.88 ( 1.62 - 2.18 ) | 1.87 ( 70.93 ) | 1.87 ( 1.65 ) | 0.9 ( 0.69 ) |
| Respiratory failure* | 167 | 4.39 ( 3.77 - 5.11 ) | 4.37 ( 431.75 ) | 4.35 ( 3.83 ) | 2.12 ( 1.9 ) |
| Dehydration* | 165 | 2.53 ( 2.17 - 2.95 ) | 2.52 ( 151 ) | 2.51 ( 2.21 ) | 1.33 ( 1.11 ) |
| Platelet count decreased* | 158 | 3.17 ( 2.71 - 3.71 ) | 3.16 ( 232.31 ) | 3.15 ( 2.76 ) | 1.65 ( 1.42 ) |
| Blood creatinine increased* | 147 | 4.26 ( 3.62 - 5.01 ) | 4.24 ( 362.48 ) | 4.22 ( 3.69 ) | 2.08 ( 1.84 ) |
| Interstitial lung disease* | 146 | 8.03 ( 6.81 - 9.45 ) | 8 ( 882.76 ) | 7.91 ( 6.9 ) | 2.98 ( 2.74 ) |
| Condition aggravated | 146 | 0.85 ( 0.72 - 0.99 ) | 0.85 ( 4.12 ) | 0.85 ( 0.74 ) | -0.24 ( -0.48 ) |
| Therapy partial responder* | 145 | 23.43 ( 19.84 - 27.66 ) | 23.33 ( 2988.19 ) | 22.53 ( 19.6 ) | 4.49 ( 4.25 ) |
| Myelosuppression* | 142 | 9.63 ( 8.15 - 11.37 ) | 9.59 ( 1076.49 ) | 9.46 ( 8.23 ) | 3.24 ( 3 ) |
| Cough | 139 | 0.95 ( 0.8 - 1.12 ) | 0.95 ( 0.39 ) | 0.95 ( 0.83 ) | -0.08 ( -0.32 ) |
| Leukopenia* | 137 | 4.38 ( 3.71 - 5.19 ) | 4.37 ( 353.99 ) | 4.35 ( 3.78 ) | 2.12 ( 1.87 ) |
| Bone marrow failure* | 135 | 11.47 ( 9.67 - 13.6 ) | 11.43 ( 1261.87 ) | 11.24 ( 9.74 ) | 3.49 ( 3.24 ) |
| White blood cell count decreased* | 134 | 2.11 ( 1.78 - 2.5 ) | 2.11 ( 77.64 ) | 2.1 ( 1.82 ) | 1.07 ( 0.82 ) |
| Headache | 130 | 0.3 ( 0.26 - 0.36 ) | 0.31 ( 205.56 ) | 0.31 ( 0.27 ) | -1.7 ( -1.95 ) |
| Erythema | 128 | 1.05 ( 0.88 - 1.25 ) | 1.05 ( 0.27 ) | 1.05 ( 0.91 ) | 0.07 ( -0.19 ) |
| Oedema peripheral* | 125 | 1.85 ( 1.55 - 2.21 ) | 1.85 ( 48.77 ) | 1.85 ( 1.59 ) | 0.89 ( 0.63 ) |
| Abdominal pain | 121 | 0.76 ( 0.64 - 0.91 ) | 0.77 ( 8.75 ) | 0.77 ( 0.66 ) | -0.39 ( -0.65 ) |
| Chest pain | 118 | 0.93 ( 0.77 - 1.11 ) | 0.93 ( 0.68 ) | 0.93 ( 0.8 ) | -0.11 ( -0.37 ) |
| Weight decreased | 118 | 0.82 ( 0.68 - 0.98 ) | 0.82 ( 4.71 ) | 0.82 ( 0.7 ) | -0.29 ( -0.55 ) |
| Alanine aminotransferase increased* | 118 | 2.64 ( 2.2 - 3.17 ) | 2.64 ( 119.53 ) | 2.63 ( 2.26 ) | 1.39 ( 1.13 ) |

Abbreviation: Asterisks (*) indicate statistically significant signals in algorithm; ROR, reporting odds ratio; PRR, proportional reporting ratio; EBGM, empirical Bayesian geometric mean; EBGM05, the lower limit of the 95% CI of EBGM; IC, information component; IC025, the lower limit of the 95% CI of the IC; CI, confidence interval; PT, preferred term.

Supplementary Table S8:

Top 50 most frequent adverse events for pemetrexed at the PT level in patients aged over 65 from FAERS data

| PT | Case numbers | ROR(95%CI) | PRR(χ^2^) | EBGM(EBGM05) | IC(IC025) |
| --- | --- | --- | --- | --- | --- |
| Malignant neoplasm progression* | 815 | 11.07 ( 10.31 - 11.88 ) | 10.83 ( 7064.03 ) | 10.53 ( 9.92 ) | 3.4 ( 3.29 ) |
| Anaemia* | 747 | 4.11 ( 3.82 - 4.42 ) | 4.04 ( 1699.06 ) | 4.01 ( 3.77 ) | 2 ( 1.89 ) |
| Pancytopenia* | 703 | 14.05 ( 13.02 - 15.17 ) | 13.79 ( 8025.34 ) | 13.29 ( 12.47 ) | 3.73 ( 3.62 ) |
| Off label use* | 686 | 1.45 ( 1.35 - 1.57 ) | 1.44 ( 94.53 ) | 1.44 ( 1.35 ) | 0.53 ( 0.42 ) |
| Diarrhoea* | 619 | 1.33 ( 1.23 - 1.45 ) | 1.33 ( 50.86 ) | 1.33 ( 1.24 ) | 0.41 ( 0.29 ) |
| Neutropenia* | 591 | 6.44 ( 5.93 - 6.99 ) | 6.35 ( 2619.24 ) | 6.25 ( 5.83 ) | 2.64 ( 2.52 ) |
| Thrombocytopenia* | 591 | 6.66 ( 6.14 - 7.23 ) | 6.57 ( 2743.82 ) | 6.46 ( 6.03 ) | 2.69 ( 2.57 ) |
| Nausea* | 518 | 1.22 ( 1.12 - 1.33 ) | 1.22 ( 20.78 ) | 1.22 ( 1.13 ) | 0.29 ( 0.16 ) |
| Febrile neutropenia* | 479 | 10.34 ( 9.44 - 11.33 ) | 10.22 ( 3870.62 ) | 9.95 ( 9.21 ) | 3.31 ( 3.18 ) |
| Pyrexia* | 474 | 2.36 ( 2.16 - 2.59 ) | 2.34 ( 365.1 ) | 2.34 ( 2.16 ) | 1.22 ( 1.09 ) |
| Acute kidney injury* | 442 | 3.07 ( 2.79 - 3.37 ) | 3.04 ( 602.57 ) | 3.02 ( 2.79 ) | 1.6 ( 1.46 ) |
| Asthenia* | 432 | 1.37 ( 1.24 - 1.5 ) | 1.36 ( 42.15 ) | 1.36 ( 1.26 ) | 0.45 ( 0.31 ) |
| Pneumonia* | 422 | 1.49 ( 1.36 - 1.64 ) | 1.49 ( 67.56 ) | 1.48 ( 1.37 ) | 0.57 ( 0.43 ) |
| Fatigue | 422 | 0.94 ( 0.85 - 1.03 ) | 0.94 ( 1.7 ) | 0.94 ( 0.87 ) | -0.09 ( -0.23 ) |
| Dyspnoea | 398 | 0.93 ( 0.84 - 1.03 ) | 0.93 ( 2.13 ) | 0.93 ( 0.86 ) | -0.1 ( -0.25 ) |
| Decreased appetite* | 387 | 2 ( 1.81 - 2.21 ) | 1.99 ( 191.09 ) | 1.99 ( 1.83 ) | 0.99 ( 0.84 ) |
| General physical health deterioration* | 374 | 3.95 ( 3.57 - 4.38 ) | 3.92 ( 807.53 ) | 3.89 ( 3.57 ) | 1.96 ( 1.81 ) |
| Vomiting* | 358 | 1.42 ( 1.28 - 1.58 ) | 1.42 ( 44.03 ) | 1.42 ( 1.3 ) | 0.5 ( 0.35 ) |
| Rash* | 350 | 1.6 ( 1.44 - 1.78 ) | 1.6 ( 78.52 ) | 1.6 ( 1.46 ) | 0.67 ( 0.52 ) |
| Interstitial lung disease* | 345 | 6.15 ( 5.53 - 6.85 ) | 6.1 ( 1448.52 ) | 6.01 ( 5.5 ) | 2.59 ( 2.43 ) |
| Disease progression* | 322 | 4.06 ( 3.64 - 4.54 ) | 4.04 ( 728.29 ) | 4 ( 3.65 ) | 2 ( 1.84 ) |
| Death | 316 | 0.44 ( 0.39 - 0.49 ) | 0.44 ( 226.46 ) | 0.44 ( 0.4 ) | -1.17 ( -1.34 ) |
| Platelet count decreased* | 287 | 3.2 ( 2.84 - 3.59 ) | 3.18 ( 425.47 ) | 3.16 ( 2.86 ) | 1.66 ( 1.49 ) |
| Drug ineffective | 270 | 0.54 ( 0.48 - 0.61 ) | 0.54 ( 104.88 ) | 0.54 ( 0.49 ) | -0.88 ( -1.05 ) |
| Leukopenia* | 249 | 7.76 ( 6.84 - 8.8 ) | 7.71 ( 1423.09 ) | 7.56 ( 6.8 ) | 2.92 ( 2.73 ) |
| Dehydration* | 245 | 2 ( 1.77 - 2.27 ) | 2 ( 121.37 ) | 1.99 ( 1.79 ) | 0.99 ( 0.81 ) |
| Renal failure* | 244 | 2.38 ( 2.1 - 2.7 ) | 2.37 ( 192.47 ) | 2.36 ( 2.12 ) | 1.24 ( 1.05 ) |
| Pneumonitis* | 212 | 9.49 ( 8.27 - 10.88 ) | 9.44 ( 1556.5 ) | 9.21 ( 8.21 ) | 3.2 ( 3 ) |
| Sepsis* | 208 | 2.28 ( 1.98 - 2.61 ) | 2.27 ( 146.88 ) | 2.26 ( 2.02 ) | 1.18 ( 0.98 ) |
| White blood cell count decreased* | 205 | 2.49 ( 2.17 - 2.86 ) | 2.48 ( 180.3 ) | 2.47 ( 2.2 ) | 1.3 ( 1.1 ) |
| Septic shock* | 188 | 5.14 ( 4.44 - 5.93 ) | 5.11 ( 613.45 ) | 5.05 ( 4.48 ) | 2.34 ( 2.12 ) |
| Hypotension | 186 | 1.06 ( 0.92 - 1.23 ) | 1.06 ( 0.7 ) | 1.06 ( 0.94 ) | 0.09 ( -0.12 ) |
| Mucosal inflammation* | 181 | 11.1 ( 9.57 - 12.88 ) | 11.05 ( 1602.92 ) | 10.73 ( 9.48 ) | 3.42 ( 3.21 ) |
| Constipation | 181 | 1.14 ( 0.98 - 1.32 ) | 1.14 ( 2.96 ) | 1.14 ( 1 ) | 0.18 ( -0.03 ) |
| Neutrophil count decreased* | 176 | 5.44 ( 4.69 - 6.32 ) | 5.42 ( 624.9 ) | 5.35 ( 4.72 ) | 2.42 ( 2.2 ) |
| Pleural effusion* | 172 | 2.92 ( 2.51 - 3.39 ) | 2.91 ( 213.71 ) | 2.89 ( 2.55 ) | 1.53 ( 1.31 ) |
| Respiratory failure* | 166 | 2.51 ( 2.16 - 2.93 ) | 2.51 ( 149.3 ) | 2.49 ( 2.19 ) | 1.32 ( 1.09 ) |
| Haemoglobin decreased* | 166 | 1.71 ( 1.47 - 2 ) | 1.71 ( 48.95 ) | 1.71 ( 1.5 ) | 0.77 ( 0.55 ) |
| Atrial fibrillation* | 162 | 1.3 ( 1.12 - 1.52 ) | 1.3 ( 11.22 ) | 1.3 ( 1.14 ) | 0.38 ( 0.15 ) |
| Pulmonary embolism* | 157 | 2.6 ( 2.22 - 3.04 ) | 2.59 ( 152.41 ) | 2.58 ( 2.26 ) | 1.37 ( 1.14 ) |
| Blood creatinine increased* | 157 | 2.59 ( 2.21 - 3.03 ) | 2.58 ( 150.75 ) | 2.57 ( 2.25 ) | 1.36 ( 1.13 ) |
| Renal impairment* | 156 | 2.1 ( 1.79 - 2.46 ) | 2.1 ( 89.02 ) | 2.09 ( 1.83 ) | 1.06 ( 0.83 ) |
| Bone marrow failure* | 149 | 10.11 ( 8.59 - 11.9 ) | 10.07 ( 1182.78 ) | 9.81 ( 8.56 ) | 3.29 ( 3.05 ) |
| Tubulointerstitial nephritis* | 146 | 11.75 ( 9.96 - 13.87 ) | 11.71 ( 1382.67 ) | 11.35 ( 9.88 ) | 3.5 ( 3.26 ) |
| Colitis* | 144 | 6.03 ( 5.11 - 7.11 ) | 6.01 ( 591.27 ) | 5.92 ( 5.16 ) | 2.57 ( 2.32 ) |
| Hypokalaemia* | 137 | 3.07 ( 2.59 - 3.63 ) | 3.06 ( 188.23 ) | 3.04 ( 2.64 ) | 1.6 ( 1.36 ) |
| Neoplasm progression* | 137 | 3.93 ( 3.32 - 4.66 ) | 3.92 ( 295.22 ) | 3.89 ( 3.38 ) | 1.96 ( 1.71 ) |
| Malaise | 134 | 0.48 ( 0.41 - 0.57 ) | 0.48 ( 74.21 ) | 0.48 ( 0.42 ) | -1.04 ( -1.29 ) |
| Oedema peripheral | 131 | 1.19 ( 1 - 1.42 ) | 1.19 ( 4.02 ) | 1.19 ( 1.03 ) | 0.25 ( 0 ) |
| Myelosuppression* | 131 | 9.04 ( 7.6 - 10.75 ) | 9.01 ( 908.8 ) | 8.8 ( 7.61 ) | 3.14 ( 2.88 ) |

Abbreviation: Asterisks (*) indicate statistically significant signals in algorithm; ROR, reporting odds ratio; PRR, proportional reporting ratio; EBGM, empirical Bayesian geometric mean; EBGM05, the lower limit of the 95% CI of EBGM; IC, information component; IC025, the lower limit of the 95% CI of the IC; CI, confidence interval; PT, preferred term.

Supplementary Table S9:

Top 50 most frequent adverse events for pemetrexed excluding common medication co-usage at the PT level from FAERS data

| PT | Case numbers | ROR(95%CI) | PRR(χ^2^) | EBGM(EBGM05) | IC(IC025) |
| --- | --- | --- | --- | --- | --- |
| Deafness | 1 | 0.58 ( 0.08 - 4.11 ) | 0.58 ( 0.31 ) | 0.58 ( 0.08 ) | -0.79 ( -2.49 ) |
| Dyspnoea* | 78 | 2.08 ( 1.67 - 2.61 ) | 2.06 ( 43.11 ) | 2.06 ( 1.65 ) | 1.04 ( 0.7 ) |
| Dry eye | 4 | 1.36 ( 0.51 - 3.64 ) | 1.36 ( 0.39 ) | 1.36 ( 0.51 ) | 0.45 ( -0.95 ) |
| Drooling | 1 | 2.22 ( 0.31 - 15.77 ) | 2.22 ( 0.67 ) | 2.22 ( 0.31 ) | 1.15 ( -1.58 ) |
| Tinnitus | 1 | 0.32 ( 0.05 - 2.29 ) | 0.32 ( 1.42 ) | 0.32 ( 0.05 ) | -1.63 ( -3.08 ) |
| Anaemia* | 51 | 4.06 ( 3.08 - 5.35 ) | 4.02 ( 116.05 ) | 4.02 ( 3.05 ) | 2.01 ( 1.52 ) |
| Flushing | 11 | 1.57 ( 0.87 - 2.84 ) | 1.57 ( 2.27 ) | 1.57 ( 0.87 ) | 0.65 ( -0.25 ) |
| Coma | 1 | 0.31 ( 0.04 - 2.18 ) | 0.31 ( 1.56 ) | 0.31 ( 0.04 ) | -1.7 ( -3.13 ) |
| Surgery | 2 | 0.56 ( 0.14 - 2.23 ) | 0.56 ( 0.7 ) | 0.56 ( 0.14 ) | -0.84 ( -2.28 ) |
| Dementia | 1 | 0.56 ( 0.08 - 3.94 ) | 0.56 ( 0.36 ) | 0.56 ( 0.08 ) | -0.85 ( -2.53 ) |
| Nausea | 44 | 0.84 ( 0.62 - 1.13 ) | 0.84 ( 1.36 ) | 0.84 ( 0.62 ) | -0.25 ( -0.68 ) |
| Oedema* | 17 | 4.71 ( 2.93 - 7.59 ) | 4.7 ( 49.53 ) | 4.7 ( 2.92 ) | 2.23 ( 1.28 ) |
| Sepsis | 7 | 0.94 ( 0.45 - 1.98 ) | 0.94 ( 0.02 ) | 0.94 ( 0.45 ) | -0.09 ( -1.1 ) |
| Pyrexia* | 48 | 2.09 ( 1.57 - 2.78 ) | 2.08 ( 27.08 ) | 2.08 ( 1.56 ) | 1.06 ( 0.61 ) |
| Dry skin | 1 | 0.12 ( 0.02 - 0.84 ) | 0.12 ( 6.58 ) | 0.12 ( 0.02 ) | -3.08 ( -4.28 ) |
| Rash | 37 | 1.23 ( 0.89 - 1.7 ) | 1.23 ( 1.6 ) | 1.23 ( 0.89 ) | 0.3 ( -0.18 ) |
| Ileus | 1 | 1.31 ( 0.19 - 9.33 ) | 1.31 ( 0.08 ) | 1.31 ( 0.19 ) | 0.39 ( -1.86 ) |
| Migraine | 1 | 0.16 ( 0.02 - 1.14 ) | 0.16 ( 4.4 ) | 0.16 ( 0.02 ) | -2.64 ( -3.9 ) |
| Jaundice | 4 | 2.15 ( 0.81 - 5.74 ) | 2.15 ( 2.46 ) | 2.15 ( 0.81 ) | 1.1 ( -0.49 ) |
| Delusion | 1 | 0.97 ( 0.14 - 6.87 ) | 0.97 ( 0 ) | 0.97 ( 0.14 ) | -0.05 ( -2.07 ) |
| Dysuria | 1 | 0.4 ( 0.06 - 2.84 ) | 0.4 ( 0.9 ) | 0.4 ( 0.06 ) | -1.32 ( -2.85 ) |
| Erythema* | 26 | 1.85 ( 1.26 - 2.73 ) | 1.85 ( 10.16 ) | 1.85 ( 1.26 ) | 0.89 ( 0.28 ) |
| Cyanosis | 2 | 1.87 ( 0.47 - 7.48 ) | 1.87 ( 0.81 ) | 1.87 ( 0.47 ) | 0.9 ( -1.13 ) |
| Abacess | 1 | 0.95 ( 0.13 - 6.76 ) | 0.95 ( 0 ) | 0.95 ( 0.13 ) | -0.07 ( -2.08 ) |
| Amnesia | 1 | 0.22 ( 0.03 - 1.57 ) | 0.22 ( 2.74 ) | 0.22 ( 0.03 ) | -2.17 ( -3.5 ) |
| Lethargy | 2 | 0.51 ( 0.13 - 2.04 ) | 0.51 ( 0.94 ) | 0.51 ( 0.13 ) | -0.97 ( -2.38 ) |
| Fatigue | 62 | 1.21 ( 0.94 - 1.56 ) | 1.21 ( 2.24 ) | 1.21 ( 0.94 ) | 0.27 ( -0.1 ) |
| Covid-19 | 2 | 0.17 ( 0.04 - 0.67 ) | 0.17 ( 8.22 ) | 0.17 ( 0.04 ) | -2.57 ( -3.77 ) |
| Fracture | 2 | 1.57 ( 0.39 - 6.26 ) | 1.57 ( 0.41 ) | 1.57 ( 0.39 ) | 0.65 ( -1.27 ) |
| Hypoxia* | 7 | 3.05 ( 1.45 - 6.39 ) | 3.04 ( 9.6 ) | 3.04 ( 1.45 ) | 1.61 ( 0.26 ) |
| Blister | 2 | 0.54 ( 0.14 - 2.17 ) | 0.54 ( 0.77 ) | 0.54 ( 0.14 ) | -0.88 ( -2.31 ) |
| Ataxia | 1 | 1.23 ( 0.17 - 8.77 ) | 1.23 ( 0.04 ) | 1.23 ( 0.17 ) | 0.3 ( -1.9 ) |
| Chorea | 1 | 8.43 ( 1.19 - 59.93 ) | 8.43 ( 6.55 ) | 8.43 ( 1.19 ) | 3.08 ( -1.2 ) |
| Overdose | 1 | 0.06 ( 0.01 - 0.46 ) | 0.06 ( 13.62 ) | 0.06 ( 0.01 ) | -3.95 ( -5.09 ) |
| Pain | 20 | 0.47 ( 0.31 - 0.74 ) | 0.48 ( 11.6 ) | 0.48 ( 0.31 ) | -1.07 ( -1.66 ) |
| Illness | 2 | 0.37 ( 0.09 - 1.48 ) | 0.37 ( 2.16 ) | 0.37 ( 0.09 ) | -1.44 ( -2.76 ) |
| Shock | 1 | 0.67 ( 0.09 - 4.74 ) | 0.67 ( 0.17 ) | 0.67 ( 0.09 ) | -0.58 ( -2.36 ) |
| Lymphoma | 1 | 0.95 ( 0.13 - 6.72 ) | 0.95 ( 0 ) | 0.95 ( 0.13 ) | -0.08 ( -2.08 ) |
| Swelling | 3 | 0.4 ( 0.13 - 1.24 ) | 0.4 ( 2.7 ) | 0.4 ( 0.13 ) | -1.32 ( -2.53 ) |
| Apnoea | 2 | 3.67 ( 0.92 - 14.68 ) | 3.67 ( 3.88 ) | 3.67 ( 0.92 ) | 1.87 ( -0.71 ) |
| Fibrosis | 1 | 4.8 ( 0.68 - 34.07 ) | 4.8 ( 3 ) | 4.79 ( 0.67 ) | 2.26 ( -1.31 ) |
| Cataract | 1 | 0.26 ( 0.04 - 1.84 ) | 0.26 ( 2.12 ) | 0.26 ( 0.04 ) | -1.95 ( -3.32 ) |
| Insomnia | 1 | 0.05 ( 0.01 - 0.39 ) | 0.05 ( 16.34 ) | 0.05 ( 0.01 ) | -4.19 ( -5.31 ) |
| Lip dry | 1 | 1.9 ( 0.27 - 13.48 ) | 1.9 ( 0.42 ) | 1.9 ( 0.27 ) | 0.92 ( -1.65 ) |
| Gangrene | 1 | 2.37 ( 0.33 - 16.84 ) | 2.37 ( 0.79 ) | 2.37 ( 0.33 ) | 1.24 ( -1.55 ) |
| Gout | 1 | 0.83 ( 0.12 - 5.89 ) | 0.83 ( 0.04 ) | 0.83 ( 0.12 ) | -0.27 ( -2.18 ) |
| Vertigo | 2 | 0.48 ( 0.12 - 1.93 ) | 0.48 ( 1.11 ) | 0.48 ( 0.12 ) | -1.05 ( -2.44 ) |
| Abulia* | 1 | 16.44 ( 2.31 - 116.9 ) | 16.44 ( 14.48 ) | 16.42 ( 2.31 ) | 4.04 ( -1.13 ) |
| Subileus | 1 | 7.26 ( 1.02 - 51.59 ) | 7.26 ( 5.39 ) | 7.26 ( 1.02 ) | 2.86 ( -1.23 ) |
| Listless | 2 | 8.94 ( 2.23 - 35.76 ) | 8.93 ( 14.08 ) | 8.93 ( 2.23 ) | 3.16 ( -0.37 ) |

Abbreviation: Asterisks (*) indicate statistically significant signals in algorithm; ROR, reporting odds ratio; PRR, proportional reporting ratio; EBGM, empirical Bayesian geometric mean; EBGM05, the lower limit of the 95% CI of EBGM; IC, information component; IC025, the lower limit of the 95% CI of the IC; CI, confidence interval; PT, preferred term.
